# Deep spatial proteomics reveals a suppressive immune niche linked to immune evasion in renal cell carcinoma

**DOI:** 10.20517/cdr.2026.27

**Published:** 2026-05-19

**Authors:** Yujia Zhang, Yicheng Zhu, Zichang Liu, Haonan Li, Bingxu Zhong, Xingang Cui, Yan Kong, Hui Lu

**Affiliations:** ^1^SJTU-Yale Joint Center for Biostatistics and Data Science, State Key Laboratory of Microbial Metabolism, Joint International Research Laboratory of Metabolic and Developmental Sciences, Department of Bioinformatics and Biostatistics, School of Life Sciences and Biotechnology, Shanghai Jiao Tong University, Shanghai 200240, China.; ^2^Shanghai Immune Therapy Institute, Shanghai Jiao Tong University School of Medicine-Affiliated Renji Hospital, Shanghai 200127, China.; ^3^Department of Urology, Xinhua Hospital, Shanghai Jiao Tong University School of Medicine, Shanghai 200092, China.; ^4^National Center for Translational Medicine, Shanghai Jiao Tong University, Shanghai 200240, China.; ^5^Shanghai-Chongqing Institute of Artificial Intelligence, Chongqing 400000, China.; ^6^Shanghai Engineering Research Center for Big Data in Pediatric Precision Medicine, Shanghai Children’s Hospital, School of Medicine, Shanghai Jiao Tong University, Shanghai 200240, China.; ^7^Institute of Bioinformatics, Shanghai Academy of Experimental Medicine, Shanghai 201203, China.; ^#^These authors contributed equally.

**Keywords:** Kidney cancer, spatial biomarker, spatial proteomics, deep learning, tumor microenvironment, regulatory T cell, immune evasion

## Abstract

**Aim:** Clear cell renal cell carcinoma (ccRCC) presents an “immune paradox” where high CD8^+^ T cell infiltration correlates with poor survival and limited response to immune checkpoint blockade (ICB). Simple cell density metrics fail to capture the functional state of the immune microenvironment, suggesting an uncharacterized spatial mechanism underlying immune evasion and poor clinical outcomes. We aimed to develop an AI-driven spatial proteomics framework to decode this immunosuppressive phenotype.

**Methods:** We developed PhenoSSP, a hierarchical deep learning framework based on a Vision Transformer backbone, designed for single-cell phenotyping from 7-channel multiplex immunofluorescence (mIF) images. It was applied to 1,633 tissue microarray (TMA) cores from 834 ccRCC patients. We defined a density-normalized Spatial Interaction Score was defined to quantify FOXP3^+^ regulatory T cell (Treg) enrichment within a 30 μm radius of CD8^+^ T cells. Survival analyses were performed using Kaplan–Meier curves with log-rank tests and multivariable Cox regression models.

**Results:** PhenoSSP achieved a balanced accuracy of 71.0% and an F1-Macro of 70.7%, outperforming conventional methods. CD8^+^ T-cell density alone was not significantly associated with overall survival (OS, *P* = 0.057), whereas the Spatial Interaction Score was significantly associated with poor OS (log-rank test, *P* = 0.012) and was significantly higher in non-survivors (*P* = 0.032).

**Conclusion:** This study reveals a spatial basis for immune evasion and poor prognosis in ccRCC: the pre-existing Treg enrichment near CD8^+^ T cells, rather than the abundance of effector cells, was associated with impaired antitumor immunity. The Spatial Interaction Score serves as a candidate prognostic biomarker and provides a rationale for Treg-targeting combination strategies in patients harboring spatially defined suppressive niches.

## INTRODUCTION

The tumor immune microenvironment (TIME) has emerged as the defining determinant of clinical outcomes in the era of immunotherapy. While high infiltration of CD8^+^ cytotoxic T lymphocytes (CTLs) is widely recognized as a hallmark of antitumor immunity and a predictor of favorable prognosis in most solid tumors (e.g., melanoma, lung cancer)^[1,2]^, clear cell renal cell carcinoma (ccRCC) presents a paradoxical immune phenomenon^[3,4]^. Importantly, this counterintuitive phenomenon is not unique to ccRCC; similar “immune paradoxes” have been increasingly reported in specific cancer subtypes, such as estrogen receptor-positive (ER^+^) breast cancer, prostate cancer, and uveal melanoma^[2]^. Large-scale clinical cohorts have repeatedly confirmed that in ccRCC, elevated CD8^+^ T cell density is paradoxically associated with higher tumor grade, increased recurrence risk, and poorer overall survival (OS)^[3,5]^. This discrepancy suggests that across these paradoxical tumor subtypes, the mere abundance of effector cells is insufficient to capture the functional state of the immune system, and suggests that an additional layer of immune regulation may influence patient outcomes.

This immune paradox has direct implications for cancer drug resistance, particularly in the context of immune checkpoint blockade (ICB). Despite the high immunogenicity of ccRCC, objective response rates to PD-1/PD-L1 monotherapy remain modest, typically ranging from 25% to 30% in advanced disease, as demonstrated by landmark clinical trials such as CheckMate-025 and KEYNOTE-426^[6,7]^. The coexistence of abundant CD8^+^ T cell infiltration with limited therapeutic efficacy strongly suggests that an intrinsic, microenvironment-mediated mechanism of primary immune evasion operates within these tumors. Unlike acquired resistance that emerges during treatment, this form of immune dysfunction appears to be architecturally pre-established within the TIME prior to therapy initiation. Identifying the spatial determinants of this pre-existing immunosuppressive phenotype is therefore critical not only for prognostic stratification but also for guiding the rational design of combination immunotherapy regimens capable of overcoming immune evasion.

Recent multi-omics studies have begun to unravel the biological complexity underlying this paradox, pointing towards a state of functional exhaustion and metabolic dysfunction. Infiltrating CD8^+^ T cells in renal cell carcinoma (RCC) often exhibit a terminally exhausted phenotype, characterized by the co-expression of inhibitory receptors (e.g., PD-1, TIM-3) and sensitivity to metabolic barriers such as the CD39-adenosine axis. However, single-cell dissociation techniques destroy the spatial architecture of the tissue, leaving a critical knowledge gap: how are these dysfunctional effectors spatially organized relative to suppressive elements? Emerging evidence highlights the “cellular niche” hypothesis, suggesting that immune function is governed by local cell-cell interactions rather than global cell counts^[8,9]^. Specifically, the physical proximity between CD8^+^ T cells and regulatory T cells (Tregs) may facilitate potent suppression via proximity-dependent mechanisms (e.g., CTLA-4 mediated trans-endocytosis) or short-range cytokine deprivation^[10,11]^. Therefore, decoding the precise spatial topology of these immune subsets is essential to resolving the RCC paradox and identifying patients who require combination therapies (e.g., anti-CTLA-4) to disrupt these suppressive niches.

Despite the urgent need for spatial profiling, quantifying these subtle architectural features remains a significant computational bottleneck. Although multiplex immunofluorescence (mIF) offers the necessary single-cell resolution, accurately identifying functionally distinct yet morphologically similar subsets, particularly morphologically subtle FOXP3^+^ Tregs amidst a sea of tumor cells, is challenging^[12]^. Traditional intensity-based thresholding and “flat” deep learning classifiers often struggle with the severe class imbalance and subcellular heterogeneity inherent in tumor tissues, leading to the misidentification of rare suppressors and the loss of critical spatial information. This lack of robust, automated phenotyping tools specific for spatial proteomics has hindered the systematic validation of spatial biomarkers in large-scale clinical cohorts.

To address this, we developed PhenoSSP, a hierarchical deep learning framework designed to mimic the “coarse-to-fine” cognitive process of pathologists. By integrating a Vision Transformer (ViT) backbone, which has demonstrated superior interpretability and performance in histopathology^[13]^, with domain-adaptive learning, PhenoSSP extracts deep features to resolve subtle immune phenotypes with high precision. We applied this framework to a large-scale cohort of 1,633 quality-controlled tissue microarray (TMA) cores from 834 patients to decode the spatial rules of immune evasion. Our analysis provides a spatial framework explaining the heterogeneous prognostic impact of CD8^+^ T cells: we demonstrate that it is not the density of CD8^+^ T cells, but their enrichment within a proximal suppressive niche by Tregs, that is strongly associated with patient survival. This study defines a “suppressive proximity” biomarker capturing a spatial determinant of immune evasion, and provides a framework for identifying patients who may benefit from combination regimens to disrupt suppressive niches.

## METHODS

### Patient cohort and data acquisition

This retrospective study was approved by the institutional review board of Xinhua Hospital, Shanghai Jiao Tong University School of Medicine (approval numbers: XHEC-C-2021-145-1, XHEC-C-2023-020-1). All patients had provided written informed consent at the time of hospital admission for the use of their clinical data. De-identified data were used for analysis. The study was conducted in accordance with institutional guidelines and the principles of the Declaration of Helsinki. Patients who underwent radical or partial nephrectomy for renal tumors between January 2012 and December 2018 were retrospectively reviewed across seven tertiary medical centers in China, coordinated by the Department of Urology, Xinhua Hospital, Shanghai Jiao Tong University School of Medicine. Inclusion criteria were: (1) histologically confirmed ccRCC; (2) availability of formalin-fixed paraffin-embedded (FFPE) tissue suitable for TMA construction; and (3) complete clinical follow-up data including OS. Exclusion criteria were: (1) non-clear cell histology (*n* = 105 cores); (2) insufficient tissue quality for mIF staining or quality control failure (*n* = 95 cores); and (3) incomplete clinical or pathological records. After applying these criteria, a total of 834 patients (1,633 TMA cores across 18 batches) were included in the final analysis. All samples were obtained prior to the administration of any systemic therapy. We utilized two independent cohorts for model development and clinical validation.

#### Model development dataset (Cohort 1)

To ensure the model learned robust features across diverse tissue morphologies, we curated a development dataset derived from 20 independent ccRCC patient samples. This dataset was designed to capture inter-patient heterogeneity. From this broader pool, 5 representative tissue regions (comprising > 120,000 cells) were selected for dense, exhaustive annotation to serve as the “Gold Standard” for fine-grained evaluation, while the remaining regions contributed to the sparse training set.

#### Validation and clinical cohort (Cohort 2)

This cohort comprised TMAs representing 1,633 TMA cores from 834 patients, collected across 18 distinct batches. All patients were annotated with complete clinical follow-up and survival data. This large-scale dataset was employed for self-supervised domain adaptation of the model and for subsequent clinical survival analysis.

#### mIF staining

FFPE tissue sections were stained using a sequential 7-color mIF protocol with the following antibodies applied in order: FOXP3 (BioLegend, Cat# 320202, 1:100), PD-1 (Cell Signaling Technology, Cat# 86163, 1:100), CD3 (Abcam, Cat# ab135372, 1:200), CD8A (Cell Signaling Technology, Cat# 70306, 1:200), CD4 (Zsbio/ZSGB-BIO, Cat# ZM-0418, 1:100), and PanCK (Sigma, Cat# C2562, 1:2,000). All primary antibodies were incubated at room temperature for 30 min. Antigen retrieval was performed using AR9 buffer. 4′,6-Diamidino-2-phenylindole (DAPI) was used for nuclear counterstaining. Whole-slide images were captured using the TissueFAXS Spectra system (TissueGnostics) at 20× magnification, with a raw resolution of 0.345 μm/pixel.

### Single-cell segmentation and gold standard generation

#### Automated single-cell segmentation

For precise single-cell delineation, we employed the deep learning-based Mesmer algorithm^[12]^ to segment all mIF images in the Development Cohort. In this process, the DAPI channel was utilized to define nuclear boundaries, while the superposition of membrane markers (CD3, CD4, CD8, and PanCK) defined the cellular membrane. This approach enabled accurate identification and contour extraction for each individual cell.

#### High-performance data preprocessing

To address the I/O bottleneck inherent in training on large-scale datasets (encompassing approximately 9.6 million cells across all cohorts), we implemented an optimized preprocessing pipeline. Each extracted 64 × 64 pixel multi-channel patch was serialized and stored as an independent, binary .npy file. This strategy allowed for high-throughput random access during training, significantly accelerating the data loading process.

#### Machine learning-assisted expert annotation

To balance biological diversity with annotation precision, we designed a rigorous “Broad-to-Dense” annotation strategy. We first utilized the full set of 20 patient samples to capture the broad phenotypic variability of ccRCC. Two experienced pathologists independently performed sparse manual annotation on randomly selected cells across these images, generating a highly diverse initial training corpus (*N* = 6,746 cells). Building on this diverse baseline, we established a high-fidelity benchmark using a “Human-in-the-Loop” approach on the 5 representative high-density regions defined in Cohort 1. A Random Forest classifier, trained on the initial diverse data, generated preliminary predictions for all cells in these regions, which were then subjected to a comprehensive, cell-by-cell review and correction by pathologists. This workflow culminated in a final, fully curated Gold Standard dataset comprising a total of over 128,000 high-quality annotated cells, ensuring that the model was exposed to the phenotypic breadth of 20 patients during learning while being rigorously evaluated against a fully annotated, whole-tissue ground truth.

### The PhenoSSP model architecture

#### PhenoSSP framework design

PhenoSSP (Phenotyping cell Subtypes in Spatial Proteomics) is a hierarchical deep learning framework designed to mimic the logical flow of pathological diagnosis. The core feature extractor is a Vision Transformer^[14]^ (ViT-Small/16, ViT-S/16) backbone initialized with DINOv2^[15]^ weights to leverage self-supervised representations. The model processes 7-channel, 64 × 64 pixel single-cell patches, which are divided into 16 × 16 sub-patches and linearly projected into a sequence of tokens. To distinguish between protein channels, we incorporated sine-cosine positional embeddings as modal identifiers. A learnable class token ([CLS]) is prepended to the sequence to aggregate global features into a 384-dimensional embedding.

#### Hierarchical classification heads

To address class imbalance and mimic pathological diagnostic logic, we designed a two-stage classification head structure. Stage 1 (Coarse Classifier) utilizes a multi-layer perceptron (MLP) to categorize cells into three primary lineages: Epithelial, Immune, and Other. Stage 2 (Immune Expert Classifier) is an independent MLP head that specifically activates for cells identified as “Immune” in the first stage. This expert classifier resolves fine-grained phenotypes, including CD3^+^CD4^+^CD8^-^ T cells, CD3^+^CD4^-^CD8^+^ T cells, CD3^-^CD4^+^CD8^-^ cells, CD3^+^CD4^-^CD8^-^ T cells, and CD4^+^FOXP3^+^ Tregs. Both classification heads incorporate Batch Normalization, ReLU activation functions, and Dropout (rate = 0.5) between fully connected layers to enhance regularization and prevent overfitting.

### Two-stage domain-adaptive training strategy

#### Stage 1: domain-adaptive self-supervised pre-training

To mitigate the domain shift arising from staining variations and maximize the utility of unlabeled data, we implemented a self-supervised pre-training phase. We aggregated a massive dataset of approximately 9.6 million unlabeled cell patches extracted from 1,633 TMA cores across 18 distinct batches. Using a Masked Autoencoder (MAE)^[16]^ approach with a 75% masking ratio, the ViT backbone was trained to reconstruct missing pixels from the masked inputs (MSE loss). This phase was conducted on a 10% subset (~960,000 cells) for 50 epochs using the AdamW optimizer^[17]^ with a learning rate of 1.5 × 10^-4^, yielding a backbone highly adapted to the specific visual characteristics of mIF data.

#### Stage 2: supervised fine-tuning with differential learning rates

Following domain adaptation, the model was fine-tuned using the expert-annotated dataset described in the “Single-Cell Segmentation and Gold Standard Generation” section. The data was stratified into 80% training and 20% validation sets. We employed a differential learning rate strategy to prevent catastrophic forgetting: the backbone (with only the last two Transformer blocks unfrozen) was fine-tuned at a low learning rate (2 × 10^-6^), while the newly initialized classification heads were trained at a higher rate (1 × 10^-4^). To strictly penalize the misclassification of rare subsets (e.g., Tregs), we minimized a Class-Weighted Cross-Entropy Loss (*L_WCE_*). Let *N_c_* be the number of samples in class *c*, and *C* be the total number of classes. The weight *w_c_* for class *c* was set inversely proportional to its frequency (*w_c_* ∝ 1/*N_c_*). The loss is defined as:

**Figure:**
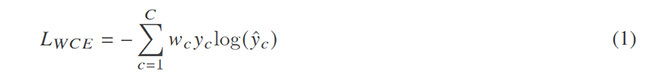


where *y_c_* is the ground truth binary indicator and 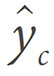 is the predicted probability. This objective function, combined with a WeightedRandomSampler, ensured that morphological features of rare cell types contributed significantly to the gradient updates. Training was governed by an early stopping mechanism, terminating if the balanced accuracy on the validation set did not improve for 5 consecutive epochs.

### Baseline models and evaluation metrics

#### Baseline models

To benchmark the performance of PhenoSSP, we compared it against three categories of models on the same 8-class classification task. First, the Flat ViT utilized the same ViT-S/16 backbone but employed a single, end-to-end classification head without the hierarchical structure. Second, the Flat convolutional neural network (CNN) employed a standard ResNet-18^[18]^ architecture trained end-to-end to represent traditional convolutional approaches. Third, an Intensity-based Machine Learning approach was implemented, where 63 handcrafted features (including mean/max intensity per channel, morphology, and nuclear-cytoplasmic ratio) were extracted per cell and classified using support vector machines (SVM)^[19]^, random forest^[20]^, and logistic regression^[21]^ algorithms.

#### Evaluation metrics

Given the significant class imbalance inherent in biological datasets, we adopted Balanced Accuracy (the arithmetic mean of recall for each class) and F1-Macro score (the arithmetic mean of per-class F1 scores) as the primary performance metrics. These indicators provide a more unbiased assessment of the model’s ability to identify rare subsets compared to standard accuracy.

### Interpretability and visualization

#### Visualizing model attention and features

To elucidate the biological basis of the model’s decisions, we employed multiple interpretability techniques. Attention Map Visualization extracted self-attention weights from the final ViT encoder block, generating heatmaps that highlight the subcellular regions most critical for classification. Feature Space Visualization involved reducing the dimensionality of patch tokens via principal component analysis (PCA) to three components and mapping them to Red-Green-Blue (RGB) space, creating “semantic segmentation” maps that reveal learned subcellular structures (e.g., nucleus *vs.* membrane) without pixel-level supervision.

#### Saliency and correlation analysis

We further utilized Guided Backpropagation (via the Captum library)^[22]^ to generate saliency maps, identifying specific pixels contributing to model predictions. Additionally, we performed Correlation Analysis by calculating the Pearson correlation coefficient between the model’s prediction confidence for specific cell types and the mean fluorescence intensity (MFI) of corresponding markers (e.g., Treg probability *vs.* FOXP3 intensity), validating the model’s alignment with biological ground truth.

### Automated inference and deployment

#### Inference workflow

For the large-scale analysis of Cohort 2 (comprising 1,633 TMA cores from 834 patients), we implemented a fully automated inference pipeline. Raw mIF images were pre-processed and segmented using DeepCell Mesmer. For each identified cell, a 64 × 64 pixel patch was extracted, normalized, and fed into the PhenoSSP framework. The inference followed the hierarchical logic: patches were first processed by the Coarse Classifier; cells identified as “Immune” were subsequently routed to the Immune Expert Classifier for subtyping. Final predictions, along with confidence scores and morphological features, were aggregated into structured files for downstream spatial analysis.

### Statistical and spatial analysis

#### Definition of spatial interaction score

Cell densities were standardized as cells/mm^2^. To rigorously quantify the “suppressive proximity” while controlling for global infiltration variability, we defined the density-normalized Spatial Interaction Score (*S_inter_*). For a patient tissue core with area *A_total_* containing a total of *N_Treg_* regulatory T cells, the Global Treg Density (*ρ_global_*) is defined as *N_Treg_*/*A_total_*. We utilized a KD-Tree algorithm^[23]^ to identify the local neighborhood for each CD8^+^ T cell. Let *C_CD_*_8_^(^*^i^*^)^ denote the *i*-th CD8^+^ T cell in the sample. The number of Tregs within a search radius *r* (set to 30 μm) of this cell is denoted as *n_Treg_*^(^*^i^*^)^(*r*). The Interaction Score is calculated as the ratio of the average local Treg density to the global Treg density:

**Figure:**
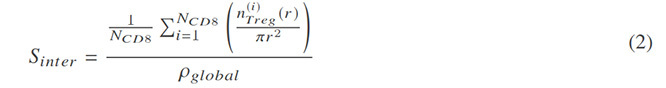


where *N_CD_*_8_ is the total number of CD8^+^ T cells in the core, and *πr*^2^ represents the area of the local circular neighborhood. A score *S_inter_* > 1 indicates active spatial enrichment (attraction), while *S_inter_* ≈ 1 suggests random distribution, and *S_inter_* < 1 implies spatial segregation (exclusion). Cores with zero Treg or zero CD8^+^ T cell counts were excluded from the Interaction Score analysis.

#### Survival and statistical analysis

The statistical significance of observed spatial patterns was assessed via permutation testing against a null distribution generated by 1,000 random permutations. For clinical correlation, OS was analyzed using Kaplan-Meier curves^[24]^ and Log-rank tests. To assess the independent prognostic value of the Interaction Score, multivariable Cox proportional hazards regression was performed using progression-free survival (PFS) as the endpoint, adjusting for age, sex, and Fuhrman grade. PFS was selected for multivariable modeling because it more directly reflects local immune control, whereas OS is additionally influenced by subsequent therapies and comorbidities. All statistical analyses were performed using Python (scipy.stats and lifelines libraries), with a two-sided *P*-value < 0.05 considered statistically significant.

#### External validation using TCGA-KIRC bulk RNA-seq data

To evaluate the generalizability of the Treg-CD8^+^ prognostic association at the bulk transcriptomic level, we analyzed an independent TCGA-KIRC cohort (*N* = 480). Immune cell fractions were estimated using the CIBERSORT algorithm^[25]^, with pre-computed infiltration scores downloaded from the TIMER3.0 database (https://compbio.cn/timer3/)^[26]^. A Treg/CD8^+^ ratio was calculated as Treg fraction/(CD8^+^ T cell fraction + 1 × 10^-6^) and patients were dichotomized at the median. Clinical data, including OS and vital status, were obtained from the Genomic Data Commons (https://portal.gdc.cancer.gov/projects/TCGA-KIRC). OS was analyzed using Kaplan-Meier curves with log-rank tests, and hazard ratios were estimated by univariate Cox proportional hazards regression.

## RESULTS

### PhenoSSP: a hierarchical framework for deep spatial feature extraction

**Figure 1:**
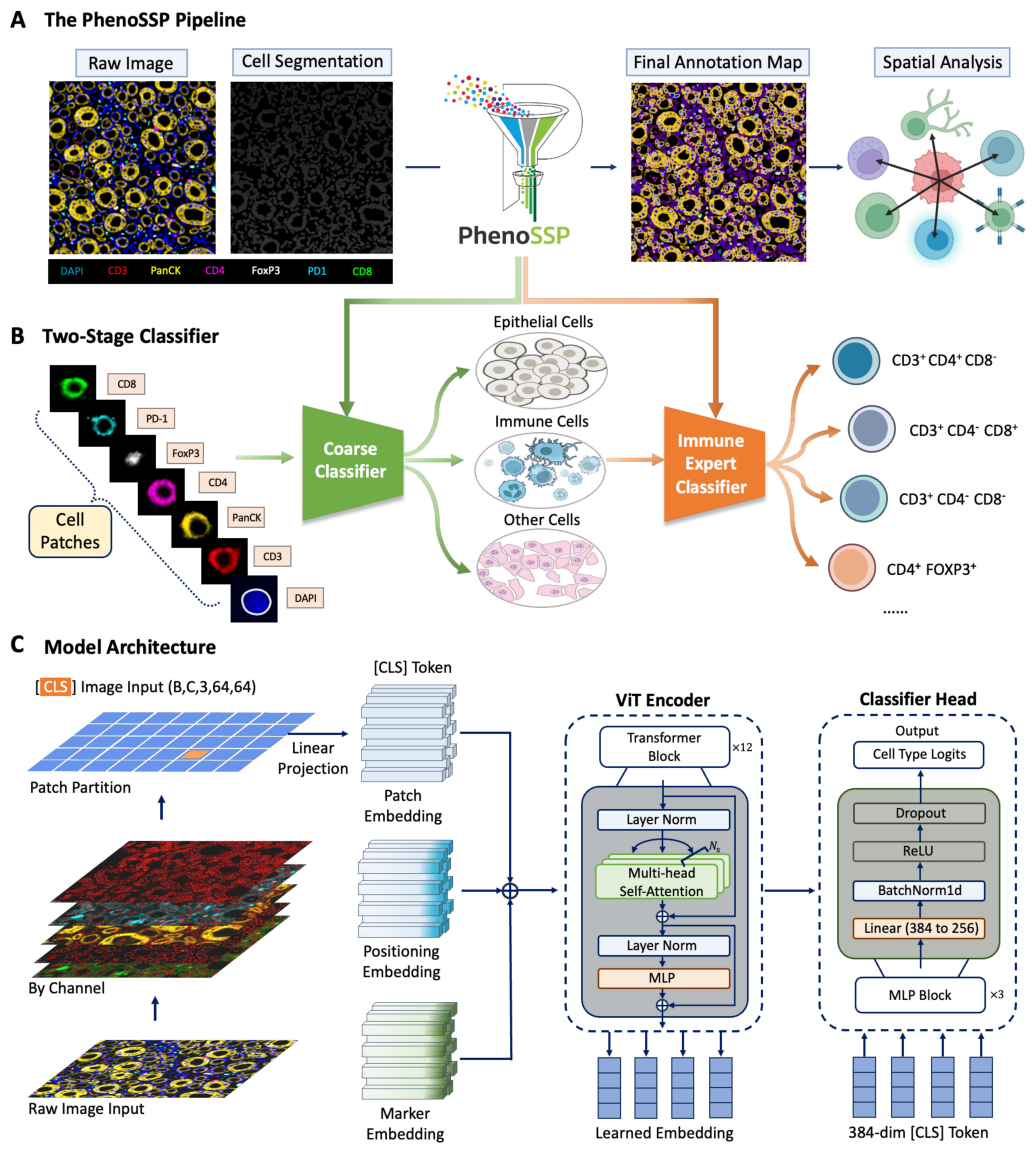
The PhenoSSP framework for deep spatial feature extraction. (A) The End-to-End Pipeline. Raw 7-color mIF images undergo cell segmentation (Mesmer) to generate single-cell patches. PhenoSSP extracts deep features to construct a final annotated spatial map, enabling downstream spatial neighborhood analysis; (B) The Hierarchical Classification Strategy. To address severe class imbalance in tissue samples, a “Coarse Classifier” first separates major lineages (Epithelial, Immune, Other), filtering out non-immune noise before an “Immune Expert Classifier” refines specific immune subtypes; (C) Model Architecture. The backbone features a ViT-S/16 initialized with DINOv2 weights. Key innovations include Marker Embeddings to handle multi-channel inputs and an MLP-based Classifier Head that processes the global [CLS] token from 64 × 64 pixel patches for precise phenotype prediction. mIF: Multiplex immunofluorescence; MLP: multi-layer perceptron; ViT: Vision Transformer.

To systematically decode the complex spatial heterogeneity of the tumor microenvironment (TME), we developed PhenoSSP, an end-to-end deep learning framework designed for high-precision single-cell phenotyping and spatial analysis [Figure 1A]. The workflow begins by processing raw 7-channel mIF images (containing DAPI, PanCK, CD3, CD4, CD8, PD-1, and FOXP3). Through cell segmentation, the tissue is discretized into individual cell-centered patches, preserving the local morphological context of each cell.

A critical challenge in TME analysis is the inherent class imbalance, where tumor and stromal cells vastly outnumber specific immune subsets. To mitigate this, we engineered a Hierarchical Classification Strategy [Figure 1B]. This cascading architecture operates in two stages: 1. Stage 1 (Coarse Classifier): Functions as a high-level filter, rapidly distinguishing cells into three major lineages: Epithelial (Tumor), Immune, and Other/Stromal. This step effectively reduces the search space and suppresses false positives from abundant non-immune cells. 2. Stage 2 (Immune Expert Classifier): Cells identified as “Immune” are routed to a specialized fine-grained classifier. This expert model focuses exclusively on resolving subtle phenotypic differences to determine specific subtypes (e.g., distinguishing CD3^+^CD4^+^CD8^-^ helper T cells from CD4^+^FOXP3^+^ Tregs).

The backbone of our framework utilizes a ViT-S/16 architecture optimized for multi-channel spatial data [Figure 1C]. Unlike standard RGB models, our architecture incorporates a specialized Marker Embedding layer alongside traditional Patch and Positioning Embeddings. This design allows the model to explicitly encode channel-specific information before processing it through the Transformer Encoder. Finally, a dedicated MLP Classifier Head projects the aggregated 384-dimensional [CLS] token into class probabilities, ensuring robust feature extraction even from noisy inputs.

### Superior performance in fine-grained immune profiling

We benchmarked PhenoSSP against a comprehensive suite of baselines, ranging from traditional machine learning algorithms (SVM, random forest) to non-hierarchical deep learning models (Flat CNN, Flat ViT).

**Figure 2:**
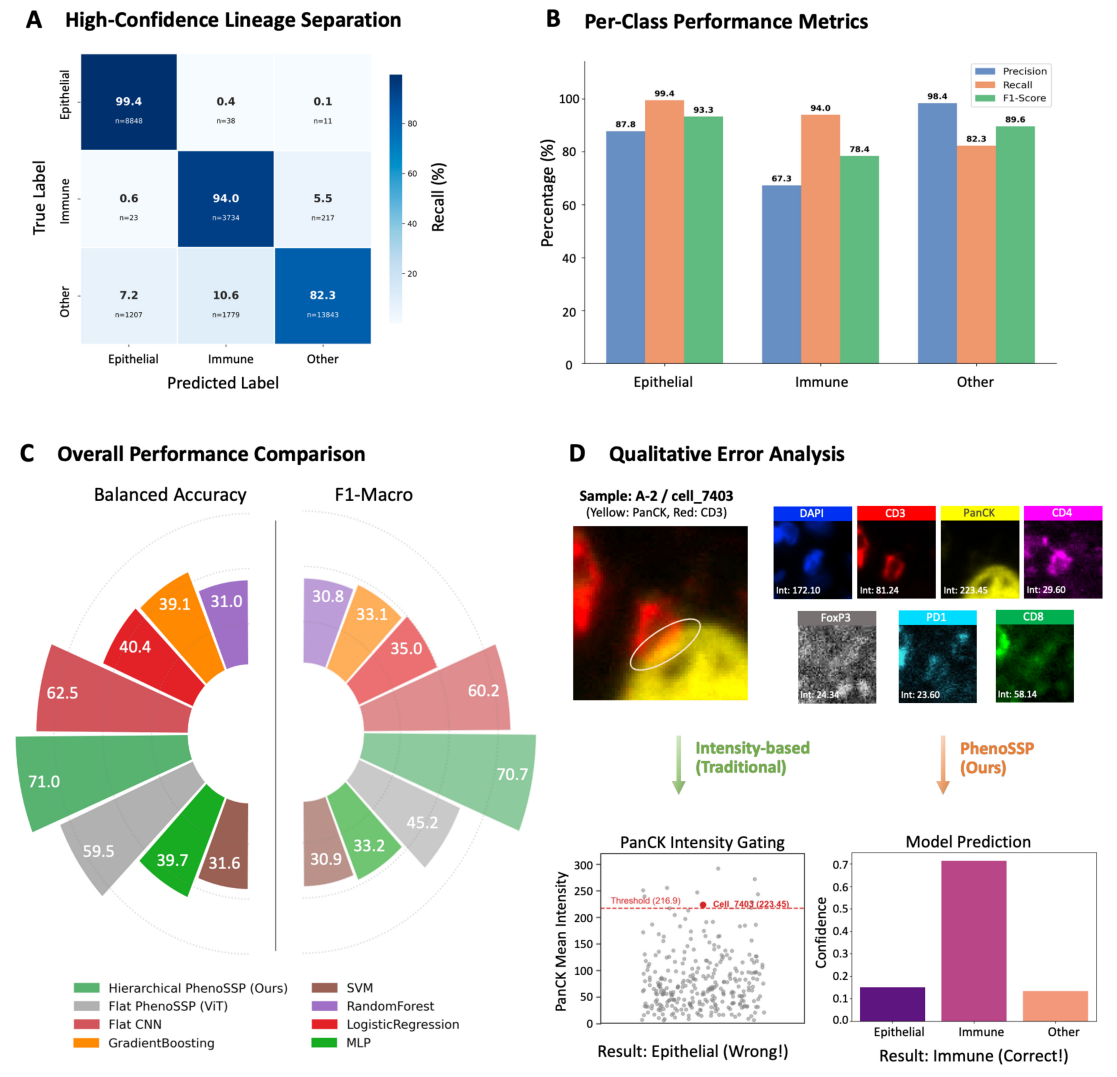
PhenoSSP demonstrates superior performance in immune phenotyping. (A) Lineage Separation Matrix. Confusion matrix of the Coarse Classifier, demonstrating near-perfect separation of Epithelial (99.4% recall) and Immune lineages (94.0% recall); (B) Per-Class Performance Metrics. Bar chart showing Precision, Recall, and F1-scores for the three coarse lineages. The model achieves a robust F1-score of 78.4% for the aggregated Immune class, laying a solid foundation for the subsequent fine-grained classification stage; (C) Overall Performance Benchmark. Circular bar plot comparing Balanced Accuracy and F1-Macro scores across different models. PhenoSSP (green) significantly outperforms Flat CNN (red) and Flat ViT (grey) architectures, while traditional machine learning methods (orange/brown) show limited capability in this complex task; (D) Qualitative Error Analysis on a “Hard Example” (Sample A-2/cell_7403). Top: Single-channel visualizations reveal an “Intensity Trap” scenario where the cell exhibits misleadingly high intensity in the PanCK channel (223.45), a marker typically exclusive to tumor cells. Bottom: Prediction confidence analysis. Despite the confounding PanCK signal, PhenoSSP correctly classifies the cell as “Immune” with high confidence (> 0.7), proving its ability to prioritize robust deep spatial features over noisy intensity values. CNN: Convolutional neural network; ViT: Vision Transformer; SVM: support vector machine; MLP: multi-layer perceptron; DAPI: 4′,6-diamidino-2-phenylindole.

The first stage of our hierarchical pipeline focuses on high-confidence lineage separation. As shown in the confusion matrix [Figure 2A], PhenoSSP achieved near-perfect classification for Epithelial cells (99.4% Recall) and robust identification of Immune cells (94.0% Recall). The per-class metrics [Figure 2B] further corroborate this stability, where the model maintained a high F1-score of 78.4% for the heterogeneous “Immune” category, successfully minimizing the leakage of immune cells into the “Other” class.

In the context of overall performance, the circular benchmark plot [Figure 2C] highlights the substantial advantage of our approach. PhenoSSP (green) achieved a Balanced Accuracy of 71.0% and an F1-Macro of 70.7%. This represents a significant performance margin over the Flat CNN (62.5% Bal Acc) and Flat ViT (59.5% Bal Acc) architectures. Notably, traditional methods like SVM and Gradient Boosting trailed by over 30%, underscoring that simple intensity features are insufficient for resolving the complex heterogeneity of the TME.

To investigate the source of this performance gain, we conducted a qualitative analysis on “hard examples” characterized by conflicting spectral signals. As illustrated in Figure 2D, we examined a representative cell (Sample A-2/cell_7403) that posed a classic “Intensity Trap”. This cell exhibited an anomalously high PanCK intensity (223.45), likely due to signal spillover or nonspecific staining, which exceeds the typical threshold for tumor cells. While a traditional intensity-based gating strategy would erroneously classify this as “Epithelial”, PhenoSSP correctly identified it as “Immune” (Ground Truth: Immune cell) with high confidence (> 0.7). This confirms that our hierarchical framework effectively transcends simple intensity thresholds by leveraging learned spatial and morphological contexts to resolve signal ambiguity.

### Robustness and visual validation

To ensure the clinical reliability of the model, we conducted a comprehensive evaluation combining qualitative “Visual Turing Tests” with quantitative benchmarking against established baselines.

**Figure 3:**
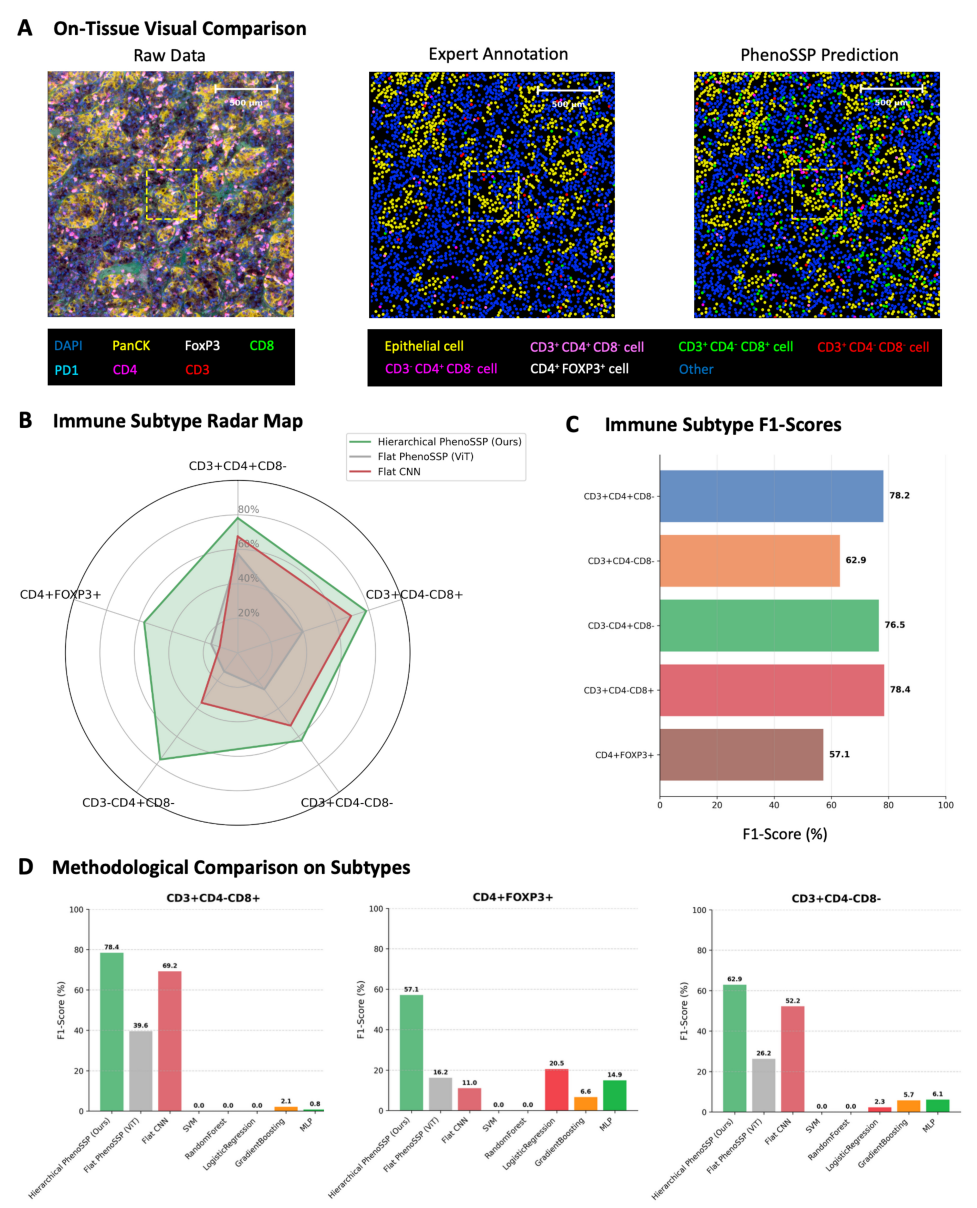
Visual and quantitative validation of model robustness. (A) On-tissue visual comparison. Phenotype maps generated by PhenoSSP (right) demonstrate high morphological fidelity and spatial concordance with Expert Annotations (center) compared to the noisy Raw Data (left). The zoomed-in regions highlight the precise localization of individual immune cells within the TME; (B) Immune Subtype Radar Map. A holistic comparison of F1-scores across five immune subtypes. The Hierarchical PhenoSSP (green area) consistently encompasses the Flat ViT and Flat CNN baselines, indicating superior performance across all categories; (C) Immune Subtype F1-scores. Bar chart detailing the specific F1-scores achieved by PhenoSSP on the held-out test set, showing strong performance (> 76%) on major T cell subsets and robust detection (> 57%) on rare Tregs; (D) Methodological Comparison on Subtypes. Comparative analysis against traditional machine learning methods (SVM, random forest, *etc.*) and flat deep learning baselines. PhenoSSP significantly outperforms traditional methods (which fail on complex multi-marker phenotypes) and shows marked improvement over Flat ViT/CNN architectures. TME: Tumor microenvironment; ViT: Vision Transformer; CNN: convolutional neural network; Tregs: regulatory T cells; SVM: support vector machine; DAPI: 4′,6-diamidino-2-phenylindole; MLP: multi-layer perceptron.

First, we assessed the model’s ability to reconstruct spatial phenotypes in dense tissue environments. As illustrated in Figure 3A, PhenoSSP generated phenotype maps that exhibited high morphological and spatial concordance with expert ground truth. Notably, the model successfully delineated the boundary between the tumor nest (Epithelial, yellow) and the stromal compartment, while accurately resolving spatially intermingled immune subsets such as cytotoxic T cells (green) and helper T cells (magenta), even in crowded regions where raw markers overlap.

Quantitatively, we evaluated the model’s performance on fine-grained immune subtyping using F1-scores. The radar chart in Figure 3B visualizes the holistic performance advantage of our hierarchical approach. PhenoSSP (green line) consistently envelops the performance polygons of both Flat ViT (grey) and Flat CNN (red) baselines, demonstrating superior sensitivity and precision across all five immune subtypes.

This performance advantage is further detailed in Figure 3C, where PhenoSSP achieved high F1-scores for distinct T cell populations, reaching 78.2% for CD3^+^CD4^+^CD8^-^ cells and 78.4% for CD3^+^CD4^-^CD8^+^ cells. Even for the most challenging phenotype, CD4^+^FOXP3^+^ Tregs, which are often obscured by low signal-to-noise ratios, the model maintained a robust F1-score of 57.1%, significantly outperforming baseline fluctuations.

Finally, we performed a rigorous methodological comparison against traditional machine learning algorithms (SVM, random forest, logistic regression) and flat deep learning models [Figure 3D]. While traditional methods struggled to identify complex phenotypes (e.g., failing to detect CD3^+^CD4^-^CD8^+^ cells with near-zero F1-scores), PhenoSSP demonstrated substantial resilience. It not only surpassed traditional ML methods by a wide margin but also outperformed the Flat ViT architecture by approximately 20%-40% in F1-scores across key subtypes. This confirms that the hierarchical incorporation of spatial constraints is critical for resolving phenotypic ambiguity that flat architectures fail to capture.

### Deep features capture biologically relevant subcellular patterns

**Figure 4:**
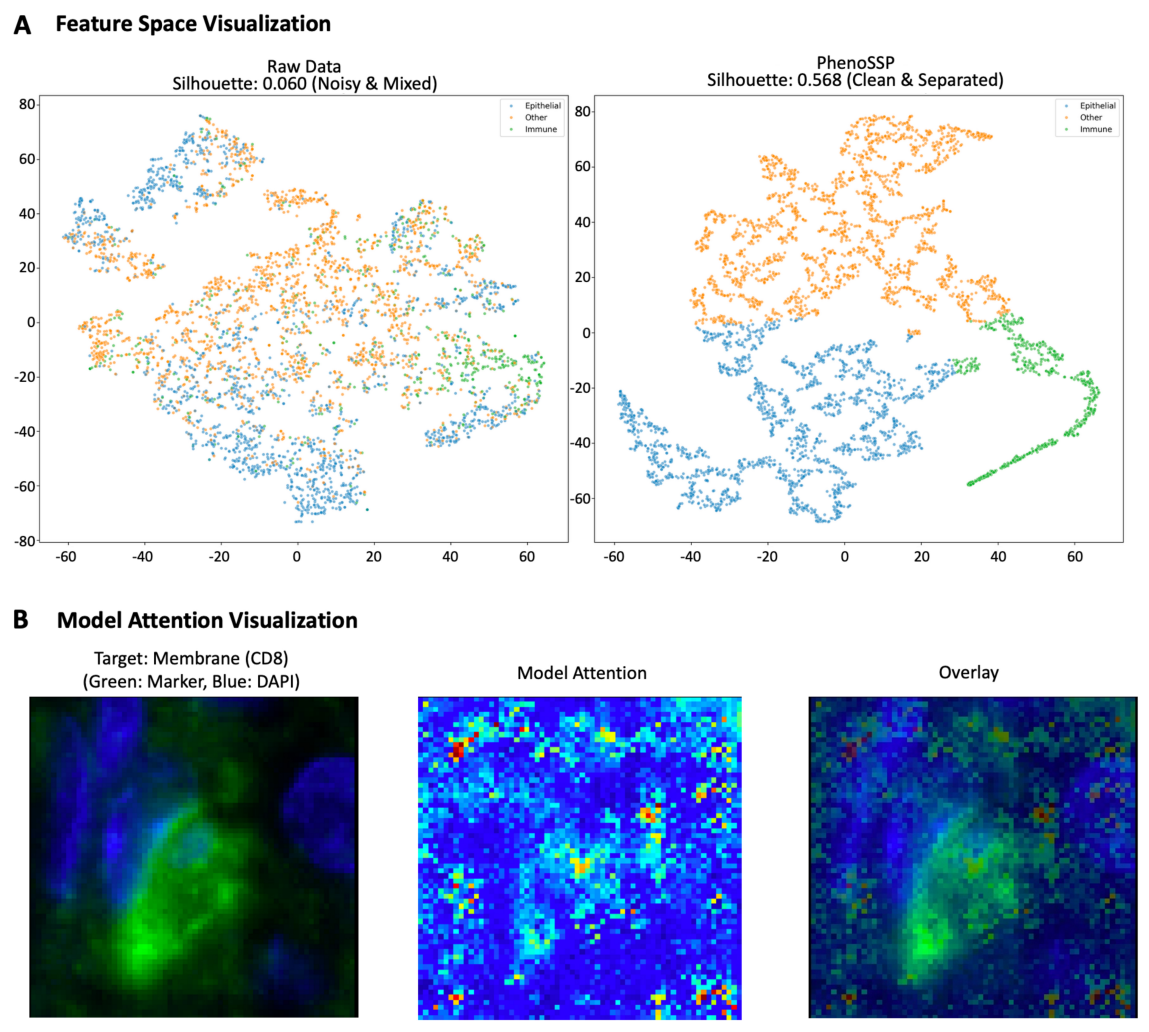
Interpretability of deep spatial features. (A) Feature space visualization (t-SNE) and clustering metrics. Left: Raw intensity features show significant overlap between Epithelial (blue), Immune (green), and Other (orange) cells with a low Silhouette score (0.060), highlighting the challenge of noise in the raw domain. Right: PhenoSSP-extracted features reveal a highly structured latent space with a significantly improved Silhouette score (0.568). The model successfully projects semantically distinct cell types into well-separated clusters, demonstrating strong discriminative power; (B) Saliency map visualization of subcellular localization. The heatmap displays pixel-wise importance derived from input gradients. For the representative CD8^+^ T cell shown, the model’s attention (red/yellow) forms a ring structure that precisely aligns with the cell membrane (green marker) and surrounds the nucleus (blue), demonstrating that the model correctly leverages membrane-bound protein signals for classification. t-SNE: t-distributed stochastic neighbor embedding; DAPI: 4′,6-diamidino-2-phenylindole.

A key advantage of PhenoSSP is its ability to learn robust, interpretable representations from noisy inputs. To evaluate this, we visualized the high-dimensional feature space using t-distributed stochastic neighbor embedding (t-SNE) to compare the raw pixel intensity data against the deep features extracted by PhenoSSP [Figure 4A].

As shown in the visualization, the raw intensity data resulted in a diffuse, highly overlapping distribution with a low Silhouette score of 0.060, confirming that pixel intensity alone is insufficient to distinguish complex cell phenotypes in the presence of background noise. In stark contrast, the PhenoSSP latent space demonstrated a nearly 10-fold improvement in cluster separability, achieving a Silhouette score of 0.568. Notably, the Immune population (green), which was previously entangled with the Epithelial (blue) and Other (orange) classes, emerged as a distinct, tightly grouped cluster in the PhenoSSP space. This quantitative and qualitative improvement indicates that the model successfully disentangles semantic biological identity from low-level background artifacts.

Furthermore, to understand the morphological basis of these decisions, we generated pixel-level saliency maps by computing the gradient of the predicted class score with respect to the input image [Figure 4B]. This analysis reveals that the model automatically learns to focus on biologically valid subcellular structures without explicit segmentation supervision. Specifically, for CD8^+^ T cells, the model’s attention is strictly confined to the perinuclear membrane region, forming a distinct ring-like pattern that precisely co-localizes with the CD8 marker while explicitly avoiding the nuclear region (DAPI). Similarly, for FOXP3^+^ Tregs, attention is concentrated on the nuclear area. This confirms that PhenoSSP decisions are driven by specific, biologically relevant signal localization rather than background noise or confounding factors.

### PhenoSSP uncovers a proximity-dependent immunosuppressive niche

**Figure 5:**
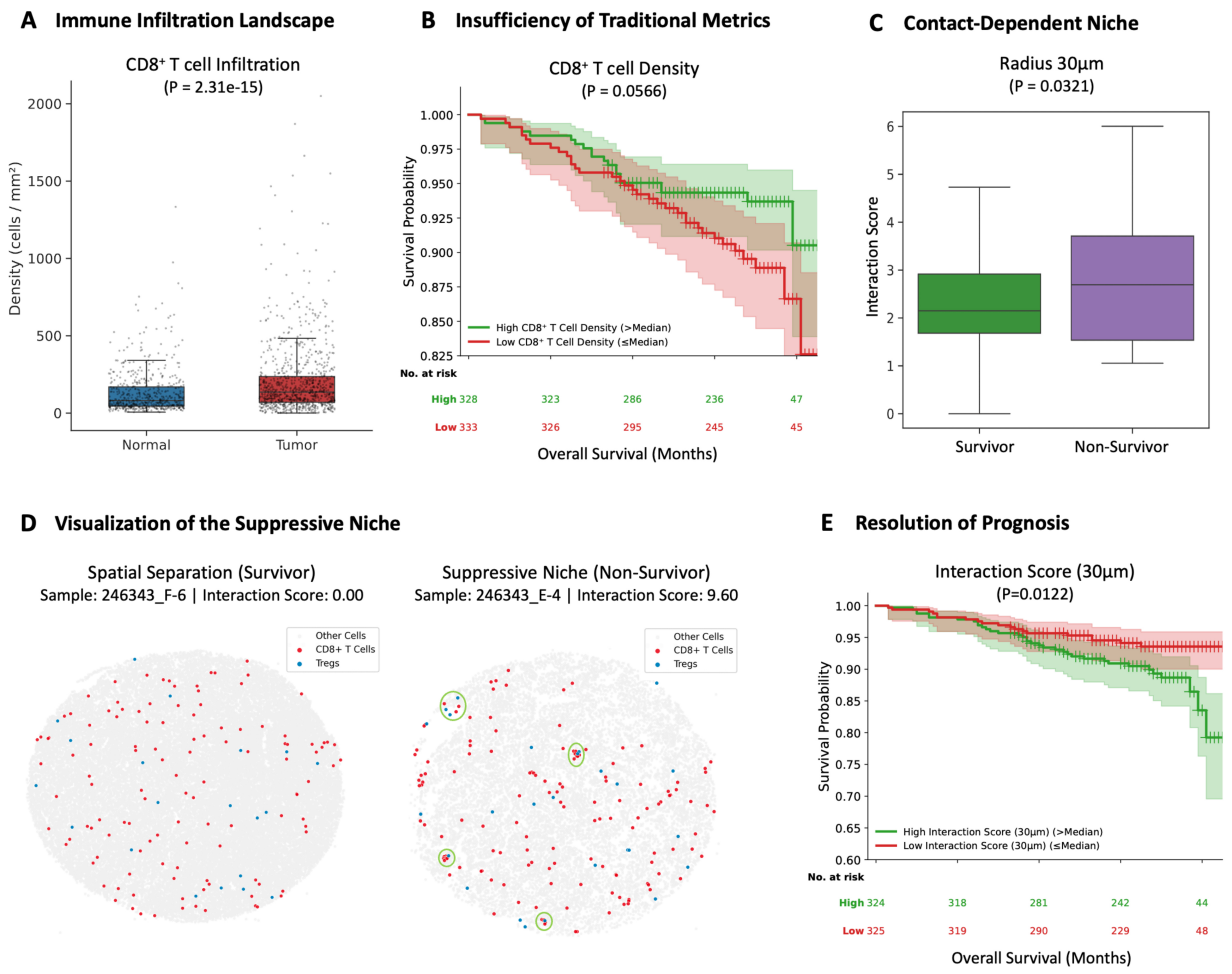
PhenoSSP reveals a proximity-dependent immunosuppressive niche that resolves the CD8^+^ T cell paradox in RCC. (A) Immune Infiltration Landscape. Quantitative analysis of cell density confirms that Tumor tissues exhibit a highly immunogenic phenotype with significantly higher CD8^+^ T cell infiltration compared to Normal tissues (P < 0.001); (B) Insufficiency of Traditional Metrics. Kaplan-Meier survival analysis reveals that CD8^+^ T cell density alone is insufficient to predict patient survival (*P* = 0.057). Although a marginal trend towards favorable prognosis was observed for high infiltration, it failed to reach statistical significance, highlighting the prognostic limitation of simple cell quantification in RCC; (C) Proximity-Dependent Niche. To investigate the mechanism of this failure, we calculated the Interaction Score (density-normalized enrichment within 30 μm). Results show that non-survivors exhibit a specific enrichment of Tregs in the immediate vicinity of CD8^+^ T cells compared to survivors (*P* = 0.032), suggesting active proximity-dependent suppression; (D) Visualization of the Suppressive Niche. Representative spatial maps of CD8^+^ T cells (red) and Tregs (blue). Left (Survivor, Sample 246343_F-6): Displays a “cold” interaction phenotype (Score = 0.00), characterized by the distinct spatial separation of effector and suppressor cells. Right (Non-Survivor, Sample 246343_E-4): Exhibits a dense “suppressive niche” (Score = 9.60). Green circles highlight areas where CD8^+^ T cells are physically surrounded by Tregs, indicating an active proximity-dependent suppression mechanism; (E) Resolution of Prognosis. Incorporating the spatial dimension resolves the paradox. Patients with a high Interaction Score exhibit significantly shorter OS (*P* = 0.012), demonstrating that the spatial positioning of immune cells, rather than their absolute abundance, dictates clinical outcome. Statistical comparisons in (A) and (C) were performed using the Wilcoxon rank-sum test. Survival analyses in (B) and (E) were conducted using Kaplan-Meier estimation with the log-rank test. Patients were stratified into high and low groups using the median value as the cutoff. RCC: Renal cell carcinoma; Tregs: regulatory T cells; OS: overall survival.

To decipher the spatial architecture of immune evasion, we first quantified the global immune landscape. Consistent with the “hot” tumor phenotype of RCC, tumor tissues exhibited significantly higher CD8^+^ T cell infiltration density compared to adjacent normal tissues (Figure 5A, *P* < 0.001). However, survival analysis exposed a critical limitation in current stratification methods: simple CD8^+^ T cell density failed to serve as a statistically significant predictor of OS (Figure 5B, *P* = 0.057). While high infiltration showed a marginal trend towards better outcomes, the lack of robust significance suggests that effector abundance alone does not guarantee antitumor efficacy, implying that the functional state of these T cells may be compromised by local microenvironmental factors.

We hypothesized that the specific spatial arrangement of suppressor cells drives this dysfunction. By calculating a density-normalized Interaction Score, we found that Tregs in non-survivors were specifically enriched within a 30 μm radius of CD8^+^ T cells (Figure 5C, *P* = 0.032). Notably, this spatial attraction diminished at larger scales (50-100 μm, Supplementary Figure 1A), strongly pointing towards a proximity-dependent or short-range paracrine suppression mechanism.

Visualization of representative spatial maps confirmed this topological distinction: survivors [Figure 5D, left] displayed a spatially dispersed topology characterized by the complete absence of Tregs in the vicinity of CD8^+^ T cells (Score = 0.00). In sharp contrast, non-survivors [Figure 5D, right] exhibited a dense “suppressive niche”, where CD8^+^ T cells were physically encircled by Tregs (Score = 9.60), creating a barrier to effective antitumor immunity.

### Spatial interaction determines clinical outcome

The clinical utility of this spatial feature was validated via Kaplan-Meier analysis. Patients with a high Spatial Interaction Score (indicating the presence of tight suppressive niches) had significantly shorter OS compared to those with spatially separated immune profiles (Log-rank test, *P* = 0.012, Figure 5E).

To verify that this metric captures true topological complexity rather than simple cell crowding, we performed rigorous correlation analyses. The Interaction Score showed only a weak negative correlation with overall cell density (Spearman R = -0.37, Supplementary Figure 1B), confirming it as an independent spatial biomarker. Furthermore, we compared our method against a naïve “nearest neighbor distance” metric. As shown in Supplementary Figure 1C, simple physical distance failed to distinguish between survivors and non-survivors (*P* = 0.4744), likely because it is confounded by global cell density variation. This validates the necessity of PhenoSSP’s density-normalized scoring algorithm.

Among conventional immune metrics, the CD8^+^/Treg ratio remained prognostic (Supplementary Figure 2A, *P* = 0.0280), whereas the absolute density of Tregs alone had no prognostic value (Supplementary Figure 2B, *P* = 0.4672). Collectively, these results demonstrate that clinical outcomes in RCC are determined not by the quantity of suppressor cells, but by their spatial positioning relative to cytotoxic effectors.

A continuous-scale analysis across 12 radii (10-150 μm) was then performed to evaluate whether the 30 μm radius represents a data-driven inflection point rather than an arbitrary threshold [Supplementary Figure 3]. The prognostic significance of the Interaction Score exhibited a clear radius-dependent pattern: statistical significance emerged at 20 μm [hazard ratio (HR) = 1.82, *P* = 0.029], peaked sharply at 35 μm (HR = 2.38, *P* = 0.002), and progressively attenuated at larger radii (HR = 1.56, *P* = 0.098 at 150 μm). This 20-35 μm significance window corresponds precisely to the biologically established range of juxtacrine signaling, including CTLA-4-mediated trans-endocytosis of co-stimulatory ligands and short-range IL-2 diffusion gradients. These results confirm that the 30 μm radius used in this study falls within an empirically validated optimal range, rather than representing an arbitrarily selected threshold.

Beyond spatial architecture, we next asked whether the Interaction Score provides independent prognostic information after accounting for established clinicopathological variables. Using PFS as the endpoint and a biologically motivated threshold (Score = 1.0), the high Interaction Score group (*n* = 152) exhibited significantly shorter PFS than the low group (*n* = 449; log-rank *P* = 0.008; Supplementary Figure 4A). In univariate Cox regression, the high group had a 2.22-fold increased risk of progression (HR = 2.22, 95%CI: 1.30-3.80, *P* = 0.004). After adjusting for age, sex, and Fuhrman grade, the Interaction Score remained an independent predictor of disease progression (HR = 1.87, 95%CI: 1.09-3.23, *P* = 0.024; Supplementary Figure 4B), confirming that the spatial metric captures prognostic information not accounted for by conventional clinical parameters.

We also examined whether CD8^+^ T cells within the suppressive niche exhibit elevated exhaustion markers by comparing PD-1 MFI inside *vs.* outside the 30 μm boundary. At the patient level, niche-resident CD8^+^ T cells exhibited statistically higher PD-1 expression (paired Wilcoxon signed-rank test, *N* = 845, *P* < 0.0001, Cohen’s d = 0.24; Supplementary Figure 5), with 489/845 (57.9%) patients showing elevated PD-1 inside the niche. However, the modest effect size (median difference of 2.3%) suggests that PD-1 upregulation alone does not fully account for the functional suppression within the niche, consistent with the hypothesis that the proximity-dependent suppressive mechanism operates primarily through PD-1-independent pathways such as CTLA-4-mediated trans-endocytosis and local IL-2 deprivation.

### Orthogonal validation of FOXP3^+^ Treg identity

A key concern is whether FOXP3^+^ cells in the ccRCC microenvironment represent true Tregs or transiently activated conventional CD4^+^ T cells. We addressed this by analyzing an independent pan-cancer CD4^+^ T cell scRNA-seq atlas^[27]^. From this atlas, we extracted 723 CD4^+^ T cells from the ccRCC subset, of which 154 were FOXP3^+^ and 569 were FOXP3^-^. FOXP3^+^ cells clustered in a spatially coherent region on UMAP that co-expressed canonical Treg signature genes, including IL2RA (CD25), CTLA4, IKZF2 (Helios), TNFRSF18 (GITR), TIGIT, and ENTPD1 (CD39) [Supplementary Figure 6A]. Dot plot analysis suggest that FOXP3^+^ cells exhibited high detection rates for all seven canonical Treg markers (76%-100%), whereas FOXP3^-^ T cells showed negligible expression (0%-19%; Supplementary Figure 6B). Quantitative comparison further confirmed that CTLA4 (90.3% *vs.* 18.5% non-zero) and IL2RA (76.0% *vs.* 12.8% non-zero) expression were markedly elevated in FOXP3^+^ cells [Supplementary Figure 6C]. These results demonstrate that FOXP3 expression in the ccRCC TME is consistently associated with a comprehensive Treg transcriptomic program, supporting the validity of the CD4^+^FOXP3^+^ definition used in our mIF-based spatial analysis.

### Cross-platform and external cohort validation

The functional relevance of the suppressive niche was further validated using an orthogonal technology platform. We analyzed a publicly available Xenium spatial transcriptomics dataset of a Stage III ccRCC specimen (10× Genomics, Xenium In Situ Gene and Protein Expression data for FFPE Human RCC; 465,534 cells, 405 genes; available at https://www.10xgenomics.com/datasets). Among 15,302 CD8^+^ T cells, 7,133 (46.6%) were located within 30 μm of a Treg - a proportion significantly exceeding the random expectation of 35.8% (permutation test, Z = 14.62, *P* < 0.001; Supplementary Figure 7A), confirming non-random spatial association.

Comprehensive gene expression profiling revealed a striking functional dichotomy between niche-resident and niche-external CD8^+^ T cells [Supplementary Figure 7B]. CD8^+^ T cells inside the niche exhibited significantly elevated expression of naive/central memory markers (IL7R/CD127, Cohen’s d = 0.51; SELL/CD62L, d = 0.47; CD28, d = 0.29) along with CTLA4 (d = 0.10), while showing significantly reduced expression of cytotoxic effector molecules (NKG7, d = -0.27; GZMA, d = -0.18; PRF1, d = -0.14; Supplementary Figure 7C and D), terminal exhaustion markers (HAVCR2/TIM-3, d = -0.28; LAG3, d = -0.19; PDCD1/PD-1, d = -0.09), and the proliferation marker MKI67 (d = -0.10). This transcriptomic profile suggests that CD8^+^ T cells within the Treg niche are maintained in an early, non-activated state rather than having undergone effector differentiation followed by suppression. The concurrent elevation of CD28 (the target of CTLA-4-mediated trans-endocytosis) and CTLA4 within the niche provides direct molecular evidence supporting the CTLA-4-dependent co-stimulation blockade mechanism proposed in this study.

Finally, we assessed cohort-level generalizability using an independent TCGA-KIRC dataset (*N* = 480). Immune cell fractions were estimated from bulk RNA-seq data using CIBERSORT (TIMER3.0) , and a Treg/CD8^+^ ratio was computed as a bulk-level proxy. Patients with a high ratio exhibited significantly shorter OS (log-rank *P* < 0.001; HR = 1.85, 95%CI: 1.37-2.52; Supplementary Figure 8). While this bulk-level proxy cannot capture the spatial proximity quantified by the Interaction Score, the directional consistency across a large, independent multi-institutional cohort provides additional support for the prognostic relevance of Treg-CD8^+^ immune balance in ccRCC.

## DISCUSSION

In this study, we addressed two related challenges in the spatial analysis of the TIME: the computational bottleneck of identifying rare cell subsets in imbalanced mIF data, and the biological enigma of the “CD8^+^ paradox” in RCC. By developing PhenoSSP, a hierarchical deep learning framework, we characterized spatial patterns associated with immune suppression, revealing that physical proximity to Tregs, rather than mere infiltration density, is a robust determinant of patient survival.

### The ccRCC immune paradox as a manifestation of microenvironment-mediated drug resistance

The prognostic failure of simple CD8^+^ T cell density in our cohort highlights the unique immunological landscape of ccRCC, which fundamentally differs from other “hot” tumors like melanoma or non-small cell lung cancer (NSCLC), where spatial heterogeneity of immune infiltration typically correlates with active antitumor responses^[28]^. While high infiltration in lung cancer often signifies an active antitumor response, ccRCC is characterized by a state of “immunogenic yet immunosuppressed” equilibrium^[29,30]^. Importantly, this paradoxical uncoupling of infiltration and cytotoxicity is not isolated to RCC; similar phenomena have been reported in specific subtypes of prostate cancer, ER^+^ breast cancer, and uveal melanoma^[2,31]^. In this context, the majority of infiltrating CD8^+^ T cells are not functional effectors. As illuminated by recent pan-cancer studies, these environments are often dominated by “bystander” CD8^+^ T cells (which recognize viral rather than tumor antigens)^[32]^ and cells trapped in a state of terminal exhaustion (e.g., CD39^+^, TIM-3^+^ phenotype)^[33-35]^.

This distinct biology explains why global metrics like tumor mutational burden (TMB), which is effective in high-mutation tumors, often fails to predict outcomes in ccRCC, a cancer type known for its relatively low mutational load but high immune infiltration^[36]^. Our findings suggest that in ccRCC, the clinical outcome is not determined by the quantity of neoantigens (TMB) or the quantity of T cells (Density), but rather by the spatial quality of the immune microenvironment. By identifying the 30 μm suppressive niche, PhenoSSP essentially distinguishes between “spatially unconstrained cells (consistent with a bystander phenotype)” and “suppressed effectors” (tumor-reactive cells actively restrained by Treg proximity).

Crucially, the identification of this 30 μm interaction radius is biologically significant. This scale corresponds precisely to the range of juxtacrine signaling and short-range diffusion (e.g., CTLA-4 mediated trans-endocytosis^[10]^) and short-range cytokine gradients (e.g., local IL-2 deprivation or TGF-β signaling^[11]^). This suggests that in high-risk patients, Tregs are not merely present but are actively recruited to the immediate vicinity of effector T cells to exert potent suppression via juxtacrine signaling. Thus, the “CD8^+^ paradox” in ccRCC is not a failure of immune recruitment, but a failure of spatial positioning, where effector cells are physically constrained within proximity-dependent suppressive niches^[9,37]^.

### Clinical implications: targeting the suppressive niche

The spatial biomarkers identified by PhenoSSP carry potential therapeutic implications for overcoming cancer drug resistance. The finding that a pre-existing suppressive niche, defined by the physical enrichment of Tregs within 30 μm of CD8^+^ T cells, predicts poor outcomes provides a mechanistic rationale for patient-specific treatment selection. We propose three specific and testable strategies for disrupting the suppressive niche, each grounded in the spatial mechanisms revealed by our data.

First, CTLA-4 blockade may serve as a targeted approach for Treg depletion within the niche. Our data demonstrate that the suppressive niche operates at a 30 μm scale, precisely matching the functional range of CTLA-4-mediated trans-endocytosis of co-stimulatory ligands^[10]^. Anti-CTLA-4 antibodies such as ipilimumab deplete intratumoral Tregs via FcγR-dependent antibody-dependent cellular cytotoxicity^[38]^, which would physically dismantle these suppressive architectures. This rationale is consistent with the demonstrated superiority of nivolumab plus ipilimumab over sunitinib in intermediate- and poor-risk ccRCC in CheckMate-214^[39]^, and with the observation by Murakami *et al.* that Foxp3^+^PD-1^+^ Tregs form spatial niches with CD8^+^ T cells that become more immunosuppressive under PD-1 blockade alone^[37]^. Our Interaction Score could thus serve as a companion biomarker to prospectively identify patients most likely to benefit from ipilimumab-containing regimens. A testable prediction arising from this analysis is that patients with an Interaction Score above the median will derive significantly greater PFS benefit from nivolumab-ipilimumab combination *vs.* PD-1 monotherapy, compared to patients with low Interaction Scores.

Second, next-generation Treg-selective depletion agents offer additional opportunities for niche disruption. Fc-engineered anti-CTLA-4 antibodies (e.g., botensilimab) and anti-CCR8 antibodies are designed to selectively deplete intratumoral Tregs while minimizing peripheral autoimmunity^[38,40]^. Our spatial framework provides a quantitative readout to evaluate whether these agents successfully reduce local Treg density within the 30 μm niche of CD8^+^ T cells. The corresponding testable prediction is that effective niche disruption, measured as a post-treatment Interaction Score below 1, will correlate with CD8^+^ T cell functional reactivation and objective tumor response.

Third, cytokine-based niche remodeling represents a complementary strategy. The 30 μm radius corresponds to the diffusion range of IL-2, a cytokine locally consumed by Tregs to suppress CD8^+^ T cell function^[11]^. IL-2 variants engineered for preferential CD8^+^ T cell binding, such as bempegaldesleukin (NKTR-214), could specifically counteract this proximity-dependent suppression by restoring local IL-2 signaling within the niche^[41]^. The testable prediction is that IL-2 variant therapy combined with PD-1 blockade will preferentially benefit patients with high Interaction Scores by rescuing cytokine-deprived CD8^+^ T cells within suppressive niches.

Importantly, our data also suggest that not all patients require niche disruption. Tumors with high CD8^+^ infiltration but low Treg proximity (low Interaction Score) may harbor spatially unconstrained, potentially functional effector cells that could respond to PD-1 monotherapy or tyrosine kinase inhibitor (TKI) regimens targeting angiogenesis. This aligns with genomic studies showing that *PBRM1* mutations are associated with angiogenesis-driven but less immunosuppressive environments, whereas *BAP1* mutations often drive an inflamed but highly suppressive phenotype^[42,43]^. The Interaction Score may therefore enable a spatial stratification framework: high-score patients directed toward Treg-targeting combinations, and low-score patients toward PD-1 monotherapy or anti-angiogenic strategies.

### The suppressive niche as a spatial mechanism of primary resistance: a hypothesis

The identification of a proximity-dependent suppressive niche provides a candidate spatial framework for understanding why a substantial proportion of ccRCC patients may exhibit primary resistance to PD-1/PD-L1 monotherapy despite harboring an immunologically “hot” phenotype^[6]^. We propose that the 30 μm suppressive niche represents a candidate structural unit of immune resistance that may operate through mechanisms orthogonal to the PD-1/PD-L1 axis.

Within this radius, Tregs exert potent suppression through at least three proximity-dependent mechanisms that PD-1 blockade alone cannot overcome: (i) CTLA-4-mediated trans-endocytosis of CD80/CD86 from antigen-presenting cells, depriving CD8^+^ T cells of essential co-stimulatory signals^[10]^; (ii) local IL-2 consumption, creating a cytokine-deprived microenvironment that impairs CD8^+^ T cell survival and proliferation even after checkpoint release^[11]^; and (iii) short-range TGF-β signaling, which drives the terminal exhaustion program in neighboring CD8^+^ T cells^[44]^. Critically, all three mechanisms are contact-dependent or short-range paracrine effects, explaining why the suppressive signal is restricted to the 30 μm radius and diminishes at larger scales (50-100 μm, Supplementary Figure 1A).

Consistent with this interpretation, our post hoc analysis of PD-1 expression within the niche revealed only a modest elevation at the protein level (Cohen’s d = 0.24, Supplementary Figure 5), indicating that the proximity-dependent suppression is not primarily mediated through the PD-1/PD-L1 axis. This finding provides a mechanistic explanation for why PD-1 blockade alone is insufficient to overcome the suppressive niche, and strengthens the rationale for targeting PD-1-independent mechanisms, particularly CTLA-4-mediated trans-endocytosis and local IL-2 deprivation, in patients harboring these spatial architectures.

The Xenium spatial transcriptomic analysis further refined our mechanistic understanding: CD8^+^ T cells within the niche retained a naive/memory-like transcriptomic signature (high IL7R, SELL, CD28; low GZMA, PRF1) rather than an effector-to-exhausted trajectory [Supplementary Figure 7B]. This suggests that Treg proximity does not merely suppress pre-existing effector function but actively prevents the initiation of the cytotoxic program, consistent with a model in which CTLA-4-mediated depletion of co-stimulatory ligands intercepts T cell priming at its earliest stage.

This spatial perspective reframes the “CD8^+^ paradox” as a potential drug resistance phenotype rather than merely a prognostic curiosity. In patients with high Interaction Scores, the pre-existing spatial architecture of suppression may constitute a structural barrier that limits the efficacy of PD-1 blockade: even when PD-1/PD-L1 engagement is disrupted, CD8^+^ T cells remain functionally constrained by the surrounding Treg niche. This is consistent with clinical observations that ccRCC patients with inflamed but immunosuppressive microenvironments often exhibit primary resistance to ICB monotherapy^[5]^, and with the finding by Murakami *et al.* that Foxp3^+^PD-1^+^ Treg niches become more immunosuppressive under PD-1 blockade^[37]^. Our data are thus consistent with the concept that the TME may architecturally pre-establish conditions unfavorable for immunotherapy efficacy prior to treatment initiation, although direct validation in ICB-treated cohorts remains essential.

### Limitations and future directions

A critical distinction must be made between prognostic and predictive biomarkers. Our entire cohort (*N* = 834) comprises exclusively treatment-naïve patients; therefore, the Spatial Interaction Score is established here as a prognostic biomarker associated with OS, not a validated predictive biomarker for response to ICB. While the biological rationale linking the suppressive niche to ICB resistance is compelling, direct demonstration of predictive utility requires validation in ICB-treated cohorts. Specifically, retrospective analysis of pre-treatment biopsies from CheckMate-214 or KEYNOTE-426 trial participants would provide an ideal testing ground. All discussion of immunotherapy resistance in this manuscript should therefore be interpreted as hypothesis-generating rather than as established clinical evidence.

Second, our definition of Tregs relies on CD4^+^FOXP3^+^ co-expression. Although FOXP3 is the canonical Treg marker, transient FOXP3 expression can occur in activated conventional CD4^+^ T cells^[45]^. To address this concern, we performed orthogonal validation using an independent pan-cancer CD4^+^ T cell scRNA-seq atlas^[27]^, demonstrating that FOXP3^+^ cells in the ccRCC microenvironment co-express a comprehensive panel of canonical Treg markers (IL2RA, CTLA4, IKZF2, TNFRSF18, TIGIT, ENTPD1), confirming their identity as true Tregs [Supplementary Figure 6]. Nevertheless, the inclusion of additional markers such as Helios and CD25 in future mIF panels would enable more precise Treg functional subtyping.

Third, our post hoc analysis of PD-1 expression within the niche revealed only a modest protein-level elevation (Cohen’s d = 0.24; Supplementary Figure 5), and the Xenium spatial transcriptomic analysis further showed that PDCD1 transcript levels were paradoxically lower inside the niche (Cohen’s d = -0.09; Supplementary Figure 7). This apparent discrepancy between protein and transcript levels is consistent with the known post-transcriptional regulation of PD-1: PD-1 protein has a long surface half-life and accumulates on the membrane even after transcriptional downregulation, whereas PDCD1 mRNA reflects the current transcriptional state of the cell^[46]^. The naive/memory-like transcriptomic profile of niche-resident CD8^+^ T cells (low PDCD1 transcription) coupled with modest protein-level retention is therefore biologically coherent and does not represent a true contradiction. While these findings support our conclusion that the suppressive mechanism is PD-1-independent, they also highlight that PD-1 was not incorporated into the Interaction Score itself. Future iterations of PhenoSSP that integrate PD-1 status into the scoring framework may enable a more granular characterization of effector cell functional states and help distinguish exhausted from bystander CD8^+^ T cells within the niche.

Fourth, our primary spatial analysis is derived from a cohort processed on a single mIF platform. To partially address this, we performed cross-platform validation using an independent Xenium spatial transcriptomics dataset (10× Genomics; 465,534 cells), which confirmed the non-random Treg-CD8^+^ spatial enrichment (permutation test, Z = 14.62, *P* < 0.001) and revealed a coherent transcriptomic signature of niche-resident CD8^+^ T cells [Supplementary Figure 7]. Nevertheless, multi-center mIF-based validation remains the foremost priority for establishing clinical generalizability. The large scale of our cohort (834 patients, 1,633 cores, 18 batches) and the cross-platform biological consistency provide a solid foundation for prospective clinical translation.

Fifth, while the saliency maps and feature space visualizations presented in this study provide evidence that PhenoSSP attends to biologically relevant subcellular structures, the internal feature attribution mechanisms of the Transformer architecture is not fully transparent. Integrating more advanced explainability methods into PhenoSSP is a direction for future work.

Looking forward, integrating PhenoSSP with spatial transcriptomics would allow direct linkage of the suppressive niche to exhaustion gene signatures (e.g., ENTPD1, HAVCR2) and genomic drivers (BAP1, PBRM1)^[42,43]^, enabling construction of a multi-modal “Spatial-Functional Atlas” of immune evasion. Extensions to graph-based decoding of tumor evolution^[47]^ and biomechanics-driven 3D architecture inference^[48]^ could further enrich the spatial understanding of immune evasion and its potential implications for drug resistance.

In conclusion, PhenoSSP shifts the analytical focus from cell density metrics to spatial architecture characterization in the TME. By enabling the precise, AI-driven identification of rare immune subsets in spatial proteomics data, our framework revealed that the spatial enrichment of Tregs within a 30 μm radius of CD8^+^ T cells, rather than effector abundance, is a key determinant of clinical outcome in ccRCC. We hypothesize that this “suppressive proximity” signature may reflect a candidate spatial mechanism of primary resistance to immune checkpoint monotherapy: the pre-existing niche architecture functionally constrains CD8^+^ T cells through PD-1-independent mechanisms, providing a potential spatial explanation for why a subset of immunologically “hot” tumors fails to respond to PD-1 blockade. Based on these findings, the Interaction Score represents a candidate prognostic biomarker that may help identify patients who could benefit from Treg-targeting combination strategies, particularly anti-CTLA-4 therapy, to disrupt suppressive niches and restore antitumor immunity. Prospective validation in ICB-treated cohorts will be essential to establish the predictive utility of this spatial biomarker for overcoming cancer drug resistance.

## DECLARATIONS

### Availability of data and materials

The PhenoSSP source code and pre-trained model weights are publicly available at https://github.com/YukiZH/PhenoSSP under the MIT license. The clinical and imaging data contain patient information and cannot be shared publicly due to ethical and privacy restrictions under the IRB approval. Processed, de-identified spatial analysis results supporting the key findings are available from the corresponding author upon reasonable request. The scRNA-seq data used for orthogonal validation of Treg identity are publicly available from the pan-cancer T cell atlas^[27]^ (http://cancer-pku.cn:3838/PanC_T/). The Xenium spatial transcriptomics dataset used for cross-platform validation is publicly available from 10× Genomics (https://www.10xgenomics.com/datasets/xenium-protein-ffpe-human-renal-carcinoma). The TCGA-KIRC clinical data are publicly available from the Genomic Data Commons (https://portal.gdc.cancer.gov/projects/TCGA-KIRC). Pre-computed immune cell infiltration estimates were obtained from the TIMER3.0 database (https://compbio.cn/timer3/).

### AI and AI-assisted tools statement

During the preparation of this manuscript, the AI tool Gemini (version 2.5 Pro, released 2025-06-17) was used solely for language editing. The tool did not influence the study design, data collection, analysis, interpretation, or the scientific content of the work. All authors take full responsibility for the accuracy, integrity, and final content of the manuscript.

### Copyright

© The Author(s) 2026.
