# Supplementary material for: Deep spatial proteomics reveals a suppressive immune niche linked to immune evasion in renal cell carcinoma
Source: Cancer Drug Resist. 2026 May 19;9:19. doi: 10.20517/cdr.2026.27 (PMC13244267; doi:10.20517/cdr.2026.27)
Supplement: Supplementary file 1 [file cdr-9-19-SupplementaryMaterials.pdf]

Supplementary Materials

Deep spatial proteomics reveals a suppressive immune niche linked to immune evasion in renal cell carcinoma

Yujia Zhang[1,#], Yicheng Zhu[2,#], Zichang Liu[3,#], Haonan Li[1], Bingxu Zhong[1], Xingang Cui[3], Yan Kong[4,5], Hui Lu[1,4,5,6,7]

[1]SJTU-Yale Joint Center for Biostatistics and Data Science, State Key Laboratory of Microbial Metabolism, Joint International Research Laboratory of Metabolic and Developmental Sciences, Department of Bioinformatics and Biostatistics, School of Life Sciences and Biotechnology, Shanghai Jiao Tong University, Shanghai 200240, China.
[2]Shanghai Immune Therapy Institute, Shanghai Jiao Tong University School of Medicine-Affiliated Renji Hospital, Shanghai 200127, China.
[3]Department of Urology, Xinhua Hospital, Shanghai Jiao Tong University School of Medicine, Shanghai 200092, China.
[4]National Center for Translational Medicine, Shanghai Jiao Tong University, Shanghai 200240, China.
[5]Shanghai-Chongqing Institute of Artificial Intelligence, Chongqing 400000, China.
[6]Shanghai Engineering Research Center for Big Data in Pediatric Precision Medicine, Shanghai Children's Hospital, School of Medicine, Shanghai Jiao Tong University, Shanghai 200240, China.
[7]Institute of Bioinformatics, Shanghai Academy of Experimental Medicine, Shanghai 201203, China.
[#]These authors contributed equally.

Correspondence to: Prof. Hui Lu, SJTU-Yale Joint Center for Biostatistics and Data Science, State Key Laboratory of Microbial Metabolism, Joint International Research Laboratory of Metabolic and Developmental Sciences, Department of Bioinformatics and Biostatistics, School of Life Sciences and Biotechnology, Shanghai Jiao Tong University, Shanghai 200240, China. E-mail: huilu@sjtu.edu.cn; Dr. Yan Kong, National Center for Translational Medicine, Shanghai Jiao Tong University, Shanghai 200240, China. E-mail: kongyan@sjtu.edu.cn. Prof. Xingang Cui, Department of Urology, Xinhua Hospital, Shanghai Jiao Tong University School of Medicine, Shanghai 200092, China. E-mail: cuixingang@xinhuamed.com.cn

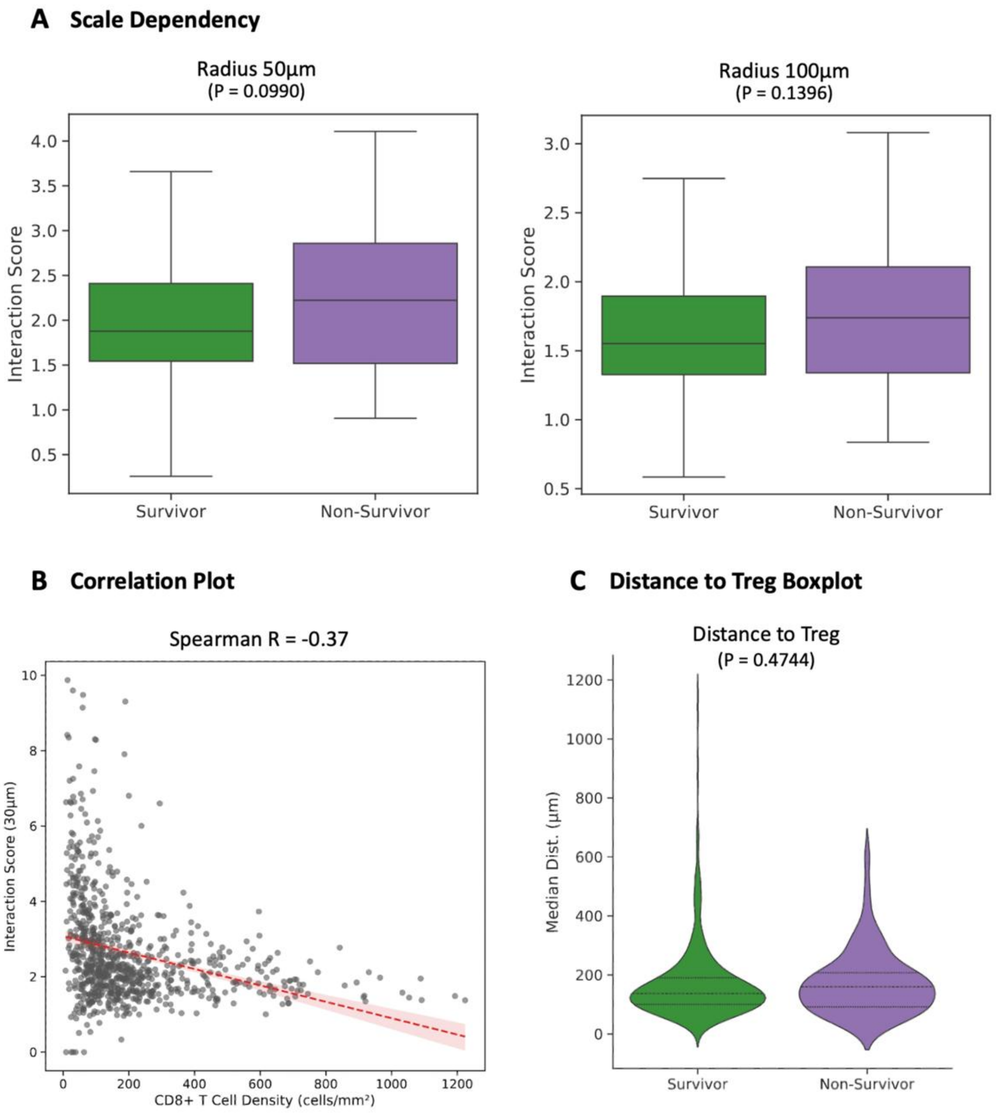

Supplementary Figure 1. Methodological validation of the Spatial Interaction Score. (A) Scale sensitivity. Boxplots comparing the Interaction Score between survivors and non-survivors at 50 µm (P = 0.099, Wilcoxon rank-sum test) and 100 µm (P = 0.140). The loss of significance at larger radii, compared to 30 µm (P = 0.032, Figure 5C), supports a proximity-dependent suppression mechanism. (B) Independence from cell density. Scatter plot of $CD8^+$ T cell density versus the Interaction Score (Spearman R = -0.37), confirming that the Score captures a distinct spatial feature independent of overall infiltration. (C) Comparison with simple distance. Violin plots of median nearest-neighbor distance from $CD8^+$ T cells to Tregs. Simple physical distance failed to distinguish survivors from non-survivors (P = 0.4744), validating the necessity of density-normalized scoring.

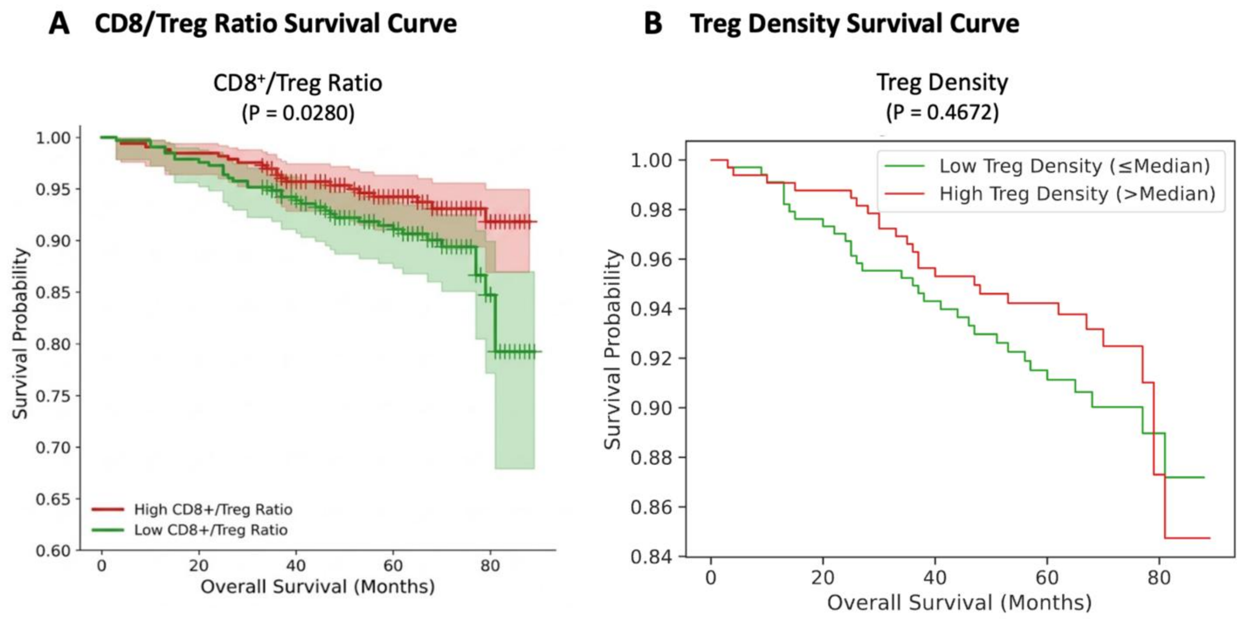

Supplementary Figure 2. Prognostic performance of conventional immune metrics. (A) $CD8^{+}$/Treg ratio. Kaplan-Meier analysis showing that a high ratio is associated with better overall survival (P = 0.028). (B) Treg density. Kaplan-Meier analysis showing that absolute Treg density alone has no prognostic value (P = 0.47). These results demonstrate that while the balance of immune cells is informative, spatial positioning provides mechanistic insight that cell counts alone cannot capture.

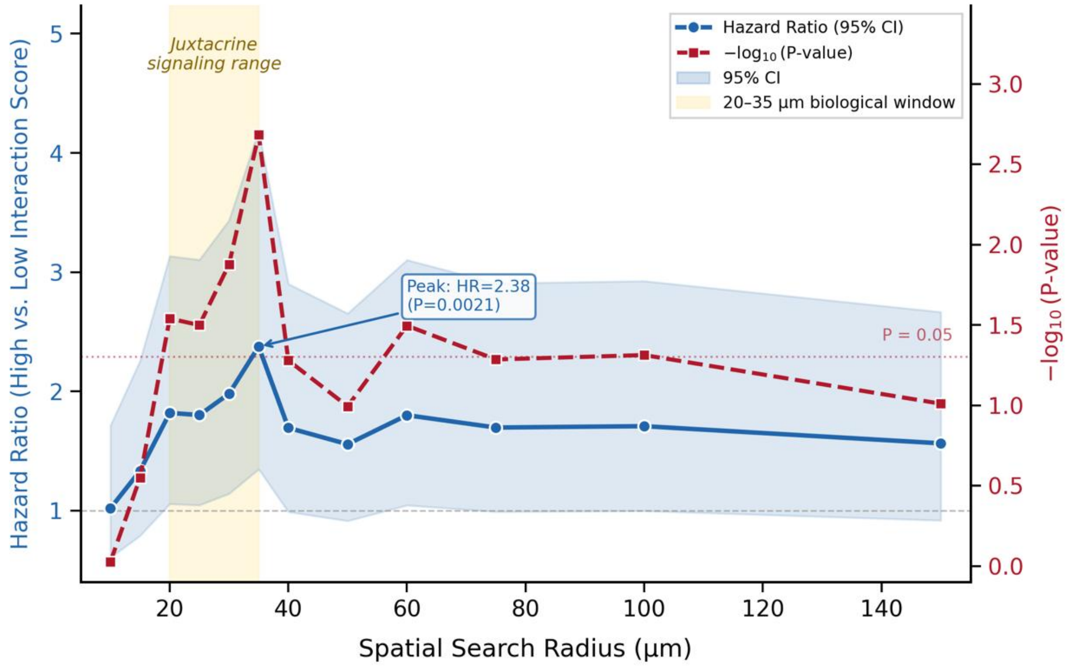

Supplementary Figure 3. Continuous-scale analysis of the Interaction Score across spatial radii. The Interaction Score was computed at 12 radii (10-150 µm) and its association with overall survival was assessed by univariate Cox regression and log-rank tests. Blue line (left axis): Hazard Ratio with 95% confidence interval. Red dashed line (right axis): $-\log_{10}$(P-value); horizontal dotted line marks P = 0.05. Yellow shading highlights the 20-35 µm biological window corresponding to juxtacrine signaling range. Peak significance was observed at 35 µm (HR = 2.38, P = 0.002).

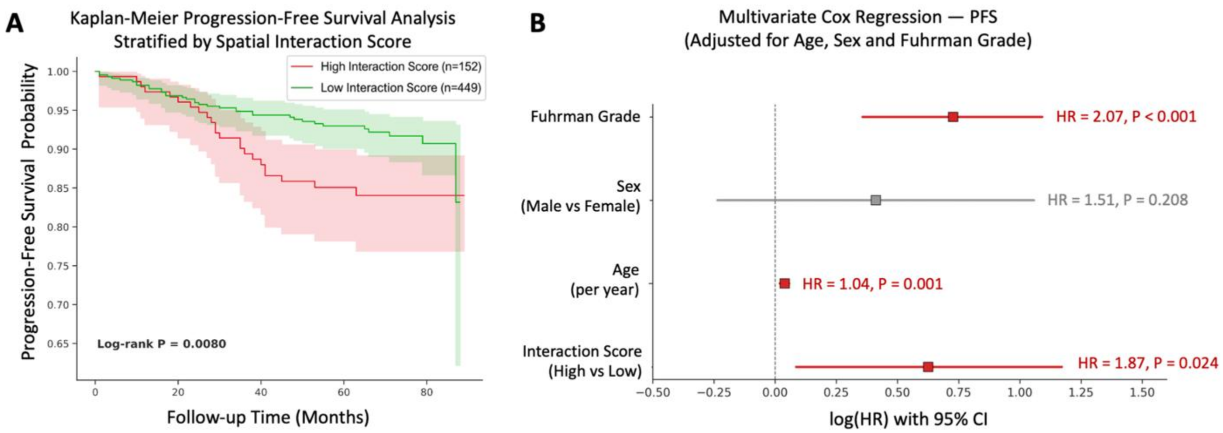

Supplementary Figure 4. Progression-free survival analysis and multivariate Cox regression of the Spatial Interaction Score. (A) Kaplan-Meier curves for PFS stratified by the Interaction Score threshold (Score = 1.0) into low (n = 449) and high (n = 152) groups. Shaded areas represent 95% confidence intervals (log-rank P = 0.008). Patients with zero Treg or zero $CD8^+$ T cell counts were excluded from this analysis. (B) Forest plot of multivariate Cox proportional hazards regression adjusted for age, sex, and Fuhrman grade. The Interaction Score remained an independent predictor of disease progression (HR = 1.87, 95% CI: 1.09 - 3.23, P = 0.024).

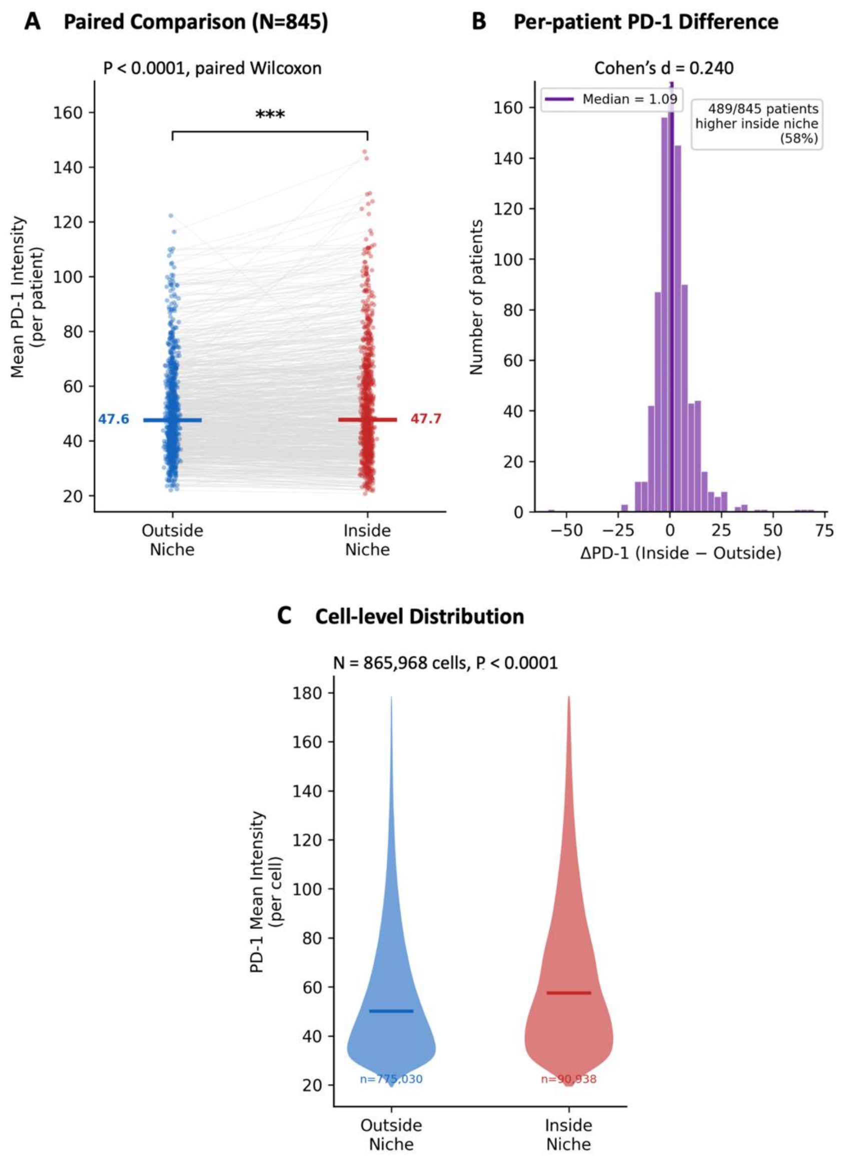

Supplementary Figure 5. PD-1 expression in $CD8^+$ T cells inside versus outside the 30 μm suppressive niche. PD-1 mean fluorescence intensity was compared between $CD8^+$ T cells located inside (≤30 μm from nearest Treg) and outside (>30 μm) the niche. (A) Paired patient-level comparison. Each line connects one patient's mean PD-1 values (N = 845; paired Wilcoxon signed-rank test, P < 0.0001). (B) Per-patient PD-1 difference distribution (ΔPD-1 = Inside - Outside). Median difference = 1.09; 489/845 (57.9%) patients showed higher PD-1 inside the niche; Cohen's d = 0.24. (C) Cell-level PD-1 distributions (outside: n = 775,030; inside: n = 90,938; Wilcoxon rank-sum test, P < 0.0001). The modest effect size suggests that PD-1 upregulation alone does not fully account for the functional suppression within the niche.

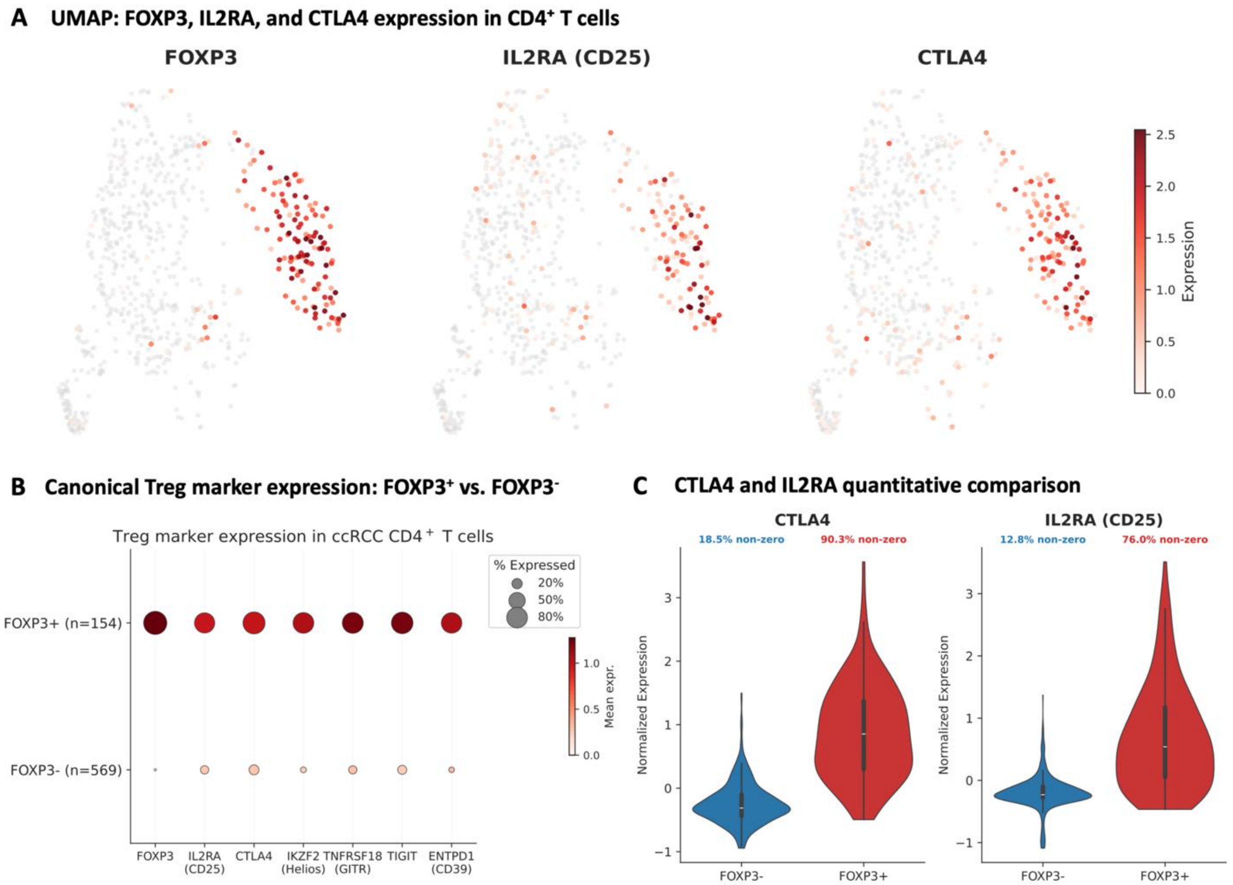

Supplementary Figure 6. Orthogonal validation of FOXP3$^+$ Treg identity by scRNA-seq. An independent pan-cancer CD4$^+$ T cell scRNA-seq atlas (Zheng et al., Science 2021) was used to validate the CD4$^+$FOXP3$^+$ Treg definition. A total of 723 CD4$^+$ T cells from the ccRCC subset were analyzed, of which 154 were FOXP3$^+$ and 569 were FOXP3$^-$. (A) UMAP colored by FOXP3 (left), IL2RA/CD25 (center), and CTLA4 (right). FOXP3$^+$ cells cluster in a spatially coherent region with co-localized Treg markers. (B) Dot plot comparing expression level (color) and detection rate (size) of seven canonical Treg markers between FOXP3$^+$ and FOXP3$^-$ cells. FOXP3$^+$ cells showed high detection rates across all markers (76-100%), whereas FOXP3$^-$ cells showed negligible expression (0-19%). (C) Violin plots of CTLA4 and IL2RA expression, confirming markedly elevated expression in FOXP3$^+$ cells (CTLA4: 90.3% vs. 18.5% non-zero; IL2RA: 76.0% vs. 12.8% non-zero). These results demonstrate that FOXP3 expression in ccRCC is consistently associated with a comprehensive regulatory T cell transcriptomic program.

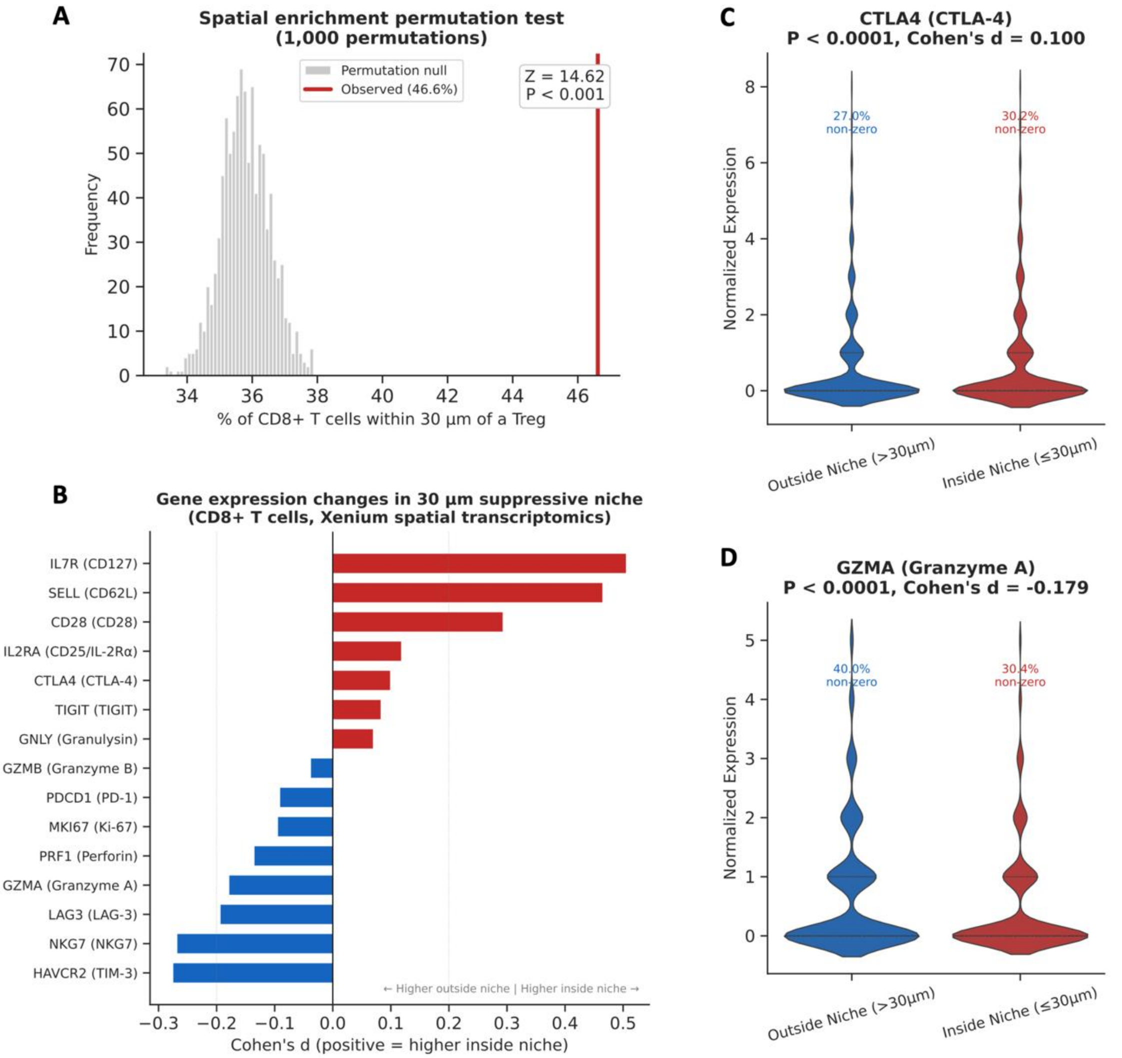

Supplementary Figure 7. Cross-platform validation of the suppressive niche by Xenium spatial transcriptomics. An independent Stage III ccRCC Xenium dataset (10x Genomics; 465,534 cells, 405 genes) was analyzed. CD8^{+} T cells (n = 15,302) were classified as Inside Niche (≤30 µm from nearest Treg; n = 7,133) or Outside Niche (>30 µm; n = 8,169). (A) Permutation test. The observed 46.6% of CD8^{+} T cells within 30 µm of a Treg far exceeded the random expectation of 35.8% (Z = 14.62, P < 0.001), confirming non-random spatial association. (B) Effect size summary of all significantly differentially expressed genes. Red: higher inside niche; blue: higher outside. Niche-resident CD8^{+} T cells exhibited a naive/memory profile (high IL7R, SELL, CD28) with elevated CTLA4, while niche-external cells displayed an effector/exhausted phenotype (high HAVCR2, NKG7, GZMA, PRF1). (C) CTLA4 violin plot. Expression was elevated inside the niche (30.2% vs. 27.0% non-zero; Cohen's d = 0.100; P < 0.0001). (D) GZMA violin plot. Expression was reduced inside the niche (30.4% vs. 40.0% non-zero; Cohen's d = −0.179; P < 0.0001), indicating impaired cytotoxic function.

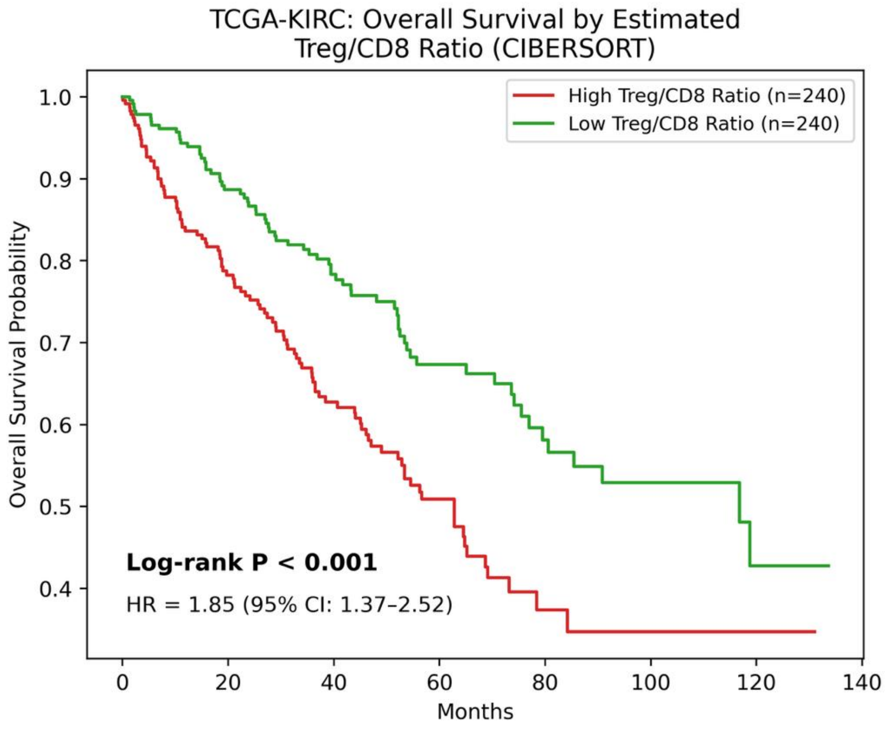

Supplementary Figure 8. External validation of the Treg/CD8$^+$ prognostic association in TCGA-KIRC. Kaplan-Meier overall survival analysis of TCGA-KIRC patients (N = 480) stratified by estimated Treg/CD8$^+$ T cell ratio. Immune cell fractions were estimated from bulk RNA-seq data using CIBERSORT (TIMER3.0 database). The Treg/CD8$^+$ ratio was calculated as Treg fraction / (CD8$^+$ T cell fraction + 1×10^{-6}) and patients were dichotomized at the median. High Treg/CD8$^+$ ratio (red, n = 240) was associated with significantly shorter overall survival compared to the low ratio group (green, n = 240; log-rank $P < 0.001$; HR = 1.85, 95% CI: 1.37–2.52, univariate Cox regression). This bulk-level analysis provides independent, directionally consistent evidence supporting the spatial observation that Treg enrichment relative to CD8$^+$ T cells is associated with poor prognosis in ccRCC, although it does not capture the spatial proximity quantified by the Interaction Score.
